# Glycemic dysregulation and insulin resistance across acute and post-acute COVID-19: a systematic review and meta-analysis of etiologic and prognostic evidence

**DOI:** 10.3389/fendo.2026.1975728

**Published:** 2026-10-14

**Authors:** José Luis Muñoz-Carrillo, Oscar Gutiérrez-Coronado, Edrei Lopez-Hernandez, Sandra Estefanía Rodríguez López, Miriam Alejandra Olivares Mendoza, Diego Octavio Ramírez Águila, Paola Trinidad Villalobos-Gutiérrez, Cesar Soria-Fregozo, Gilberto Uriel Rosas-Sánchez, Alejandro David Soriano-Hernández

**Affiliations:** 1 Laboratorio de Inmunología, Centro Universitario de los Lagos, Universidad de Guadalajara, Jalisco, Lagos de Moreno, Mexico; 2 Centro de Investigación e Innovación, Global University, Aguascalientes, Aguascalientes, Mexico; 3 Facultad de Medicina, Unidad de Ciencias de la Salud, Universidad Veracruzana, Veracruz, Xalapa de Enríquez, Mexico; 4 Ingenieria Bioquímica, Centro Universitario de los Lagos, Universidad de Guadalajara, Jalisco, Lagos de Moreno, Mexico; 5 Departamento de Ciencias de la Tierra y de la Vida, Centro Universitario de Los Lagos, Universidad de Guadalajara, Jalisco, Lagos de Moreno, Mexico; 6 Programa de Estancias Posdoctorales por Mexico, Secretaría de Ciencia, Humanidades, Tecnología e Innovación, Centro Universitario de Los Lagos, Universidad de Guadalajara, Jalisco, Lagos de Moreno, Mexico

**Keywords:** COVID-19, hyperglycemia, insulin resistance, mechanical ventilation, mortality, newly diagnosed diabetes, SARS-CoV-2, stress hyperglycemia

## Abstract

**Background:**

Coronavirus disease 2019 (COVID-19) can disrupt glucose regulation through inflammation, adipose-tissue dysfunction, impaired insulin signaling, and variable β-cell compensation. These evidence domains remain incompletely integrated.

**Objective:**

To synthesize evidence on glycemic dysregulation and insulin resistance or insulin sensitivity across acute and post-acute COVID-19, including metabolic trajectories and associations with adverse clinical outcomes.

**Methods:**

PubMed, Embase, Scopus, Web of Science, and ScienceDirect were searched. Eligible studies evaluated glucose regulation and at least one insulin-resistance or insulin-sensitivity measure during or after COVID-19. Etiological and prognostic evidence was analyzed as related but distinct domains. Prognostic meta-analysis evaluated study-defined acute hyperglycemic phenotypes; insulin-resistance or insulin-sensitivity assessment was a study-level eligibility criterion rather than part of a participant-level composite exposure. Unadjusted risk ratios (RRs) from event counts were pooled using restricted maximum-likelihood random-effects models with Hartung–Knapp adjustment. Non-combinable evidence was synthesized according to Synthesis Without Meta-analysis principles. Risk of bias was assessed using design-appropriate tools, and certainty was evaluated with GRADE. The protocol and analytical plan were retrospectively registered in OSF on August 21, 2026 (https://doi.org/10.17605/OSF.IO/K5FWU).

**Results:**

Twenty independent studies represented by 23 reports. Acute hyperglycemic phenotypes were associated with increased short-term all-cause mortality (four studies; 1,079 participants; RR 2.09, 95% confidence interval [CI] 1.21–3.64; I^2^=31.3%; 95% prediction interval 0.96–4.56) and invasive mechanical ventilation (three studies; 679 participants; RR 3.64, 95% CI 1.74–7.61; I^2^=0%). Mortality estimates remained directionally stable in sensitivity analysis. Adding a large study using peak in-hospital glucose preserved the mortality association but produced substantial heterogeneity for ventilation. Etiological evidence showed no uniform post-COVID trajectory: insulin resistance or glycemic abnormalities resolved in some populations, persisted in others, and progressed toward newly diagnosed diabetes in a subset. Mechanistic evidence implicated inflammatory stress, adipose-tissue dysfunction, impaired insulin signaling, and variable β-cell responses.

**Conclusions:**

Study-defined acute hyperglycemic phenotypes were associated with adverse COVID-19 outcomes in studies that also assessed insulin resistance or insulin sensitivity. These unadjusted estimates should not be interpreted as effects of a combined phenotype or as causal associations. Persistent metabolic abnormalities occurred heterogeneously, supporting standardized participant-level definitions and controlled longitudinal studies.

**Systematic review registration:**

https://doi.org/10.17605/OSF.IO/K5FWU, identifier 10.17605/OSF.IO/K5FWU.

## 1 Introduction

Coronavirus disease 2019 (COVID-19) is increasingly recognized as a multisystem disorder with clinically relevant metabolic manifestations. The relationship between diabetes mellitus and COVID-19 is bidirectional: pre-existing diabetes and poor glycemic control increase the probability of severe disease, whereas severe acute respiratory syndrome coronavirus 2 (SARS-CoV-2) infection and its associated inflammatory response can disrupt glucose homeostasis in individuals with and without previously diagnosed diabetes (1). Importantly, acute hyperglycemia appears to convey prognostic information beyond the presence of chronic diabetes. A meta-analysis of 16 observational studies involving 6,386 hospitalized patients found that admission hyperglycemia was associated with greater odds of mortality and severe or critical COVID-19 (2). More recently, a meta-analysis of seven studies comprising 2272 patients showed that an elevated stress hyperglycemia ratio was associated with increased risks of invasive mechanical ventilation and in-hospital mortality (3). These findings identify acute glycemic dysregulation as a clinically important feature of COVID-19, although they do not establish whether hyperglycemia is merely a marker of severe physiological stress or a marker of a broader metabolic disturbance.

The mechanisms underlying COVID-19-associated hyperglycemia are heterogeneous and potentially overlapping. Acute inflammatory signaling, increased counter-regulatory hormones, hepatic glucose production, impaired peripheral glucose uptake, oxidative stress, endothelial dysfunction, and treatment-related exposures can all disturb glucose regulation (1). Systemic glucocorticoids, particularly in severe disease, can further increase hepatic gluconeogenesis, reduce insulin sensitivity, and impair β-cell compensation, complicating attribution of hyperglycemia specifically to SARS-CoV-2 (4). Nevertheless, clinical and experimental evidence supports biologically specific effects of the infection. Human adipose-tissue studies have demonstrated productive SARS-CoV-2 infection of adipocytes and abortive infection of adipose-tissue-resident macrophages, accompanied by a local inflammatory response that provides a biologically plausible link between infection and metabolic dysfunction (5). Experimental studies using human pancreatic tissue and isolated islets have also demonstrated SARS-CoV-2 entry into β cells, reduced insulin content and secretion, and increased β-cell apoptosis, although the magnitude of this mechanism in clinical populations remains uncertain (6). Clinical observations also suggest that COVID-19-associated hyperglycemia may reflect overlapping contributions from stress physiology, insulin resistance, impaired β-cell compensation, glucocorticoid exposure, and previously unrecognized diabetes (7). Collectively, this evidence supports considering hyperglycemia and insulin resistance as related but non-equivalent metabolic abnormalities that may coexist during COVID-19.

The temporal relevance of these metabolic abnormalities may extend beyond acute infection. Post-infection hyperglycemia and newly diagnosed diabetes have been reported; however, it remains uncertain whether these abnormalities represent transient stress responses, the unmasking of pre-existing metabolic disease, or direct or indirect consequences of SARS-CoV-2 infection (7). At the population level, a systematic review and meta-analysis of 12 cohort studies involving more than 48 million participants reported a 41% higher relative risk of new-onset diabetes after COVID-19 compared with non-infected populations, with stronger associations among adults and individuals who experienced more severe acute disease (8). A subsequent systematic review restricted to adults without previous type 2 diabetes found deterioration in 16 of 25 insulin-resistance comparisons and in 7 of 12 β-cell-function comparisons following COVID-19 exposure, although measurement heterogeneity and methodological limitations constrained causal interpretation (9). A long-COVID-focused meta-analysis similarly reported higher HOMA-IR, fasting insulin, fasting and random glucose, and lower insulin sensitivity among individuals with long COVID than among control participants (10). These findings indicate that post-COVID metabolic abnormalities are clinically plausible, but their interpretation remains complicated by baseline adiposity, undiagnosed pre-existing dysglycemia, acute disease severity, corticosteroid exposure, selection of survivors, heterogeneous follow-up periods, and the absence of adequately reassessed control groups in several studies.

Previous evidence syntheses have addressed distinct portions of this metabolic continuum. Meta- analysis of admission hyperglycemia and the stress hyperglycemia ratio have concentrated on acute prognosis without requiring concurrent characterization of insulin resistance (2, 3). Reviews of incident diabetes have quantified post-infection diagnoses but have not established the metabolic pathways through which post-infection glycemic dysregulation develops (8). Conversely, systematic reviews of insulin resistance and β-cell dysfunction have focused on etiological metabolic changes in selected populations (9), while long-COVID meta-analysis has emphasized differences in individual biomarkers compared with control groups without integrating acute clinical prognosis (10). Consequently, the available literature has not established, within a single analytical framework, how SARS-CoV-2-associated insulin resistance and glycemic dysregulation evolve from acute infection into the post-acute period or whether study-defined acute hyperglycemic states, in studies also assessing insulin resistance or insulin sensitivity, identify patients at increased risk of major clinical outcomes. Moreover, pooling glucose concentrations, HOMA-derived indices, insulin-sensitivity measures, lipid-based surrogate indices, incident diabetes, and clinical outcomes into a single summary estimate would obscure rather than resolve these differences.

Accordingly, this systematic review and meta-analysis addressed two related but analytically distinct evidence domains. The etiologic domain examined relationships between SARS-CoV-2 infection, glycemic dysregulation, and insulin resistance or insulin sensitivity across acute and post-acute disease. The prognostic domain evaluated associations between study-defined acute hyperglycemic phenotypes and adverse clinical outcomes in studies that also assessed insulin resistance or insulin sensitivity. Additional objectives were to characterize changes in insulin-resistance and insulin-sensitivity indices, β-cell function, incident or progressive glycemic abnormalities and diabetes, and the inflammatory, adipose-tissue, and pancreatic mechanisms potentially connecting acute metabolic disruption with subsequent clinical and glycemic outcomes.

## 2 Materials and methods

### 2.1 Protocol registration and reporting

This systematic review and meta-analysis was conducted and reported in accordance with the Preferred Reporting Items for Systematic Reviews and Meta-Analyses 2020 (PRISMA 2020) statement (11). Reporting of the literature search was further guided by the PRISMA extension for reporting literature searches (PRISMA-S) (12), while reporting of the quantitative synthesis of etiologic and prognostic evidence from observational studies was additionally informed by the Meta-analysis of Observational Studies in Epidemiology (MOOSE) recommendations (13). The finalized protocol and analytical plan were retrospectively registered in the Open Science Framework on August 21, 2026 (OSF; registration DOI: https://doi.org/10.17605/OSF.IO/K5FWU). Registration occurred after completion of the literature search, study selection, standardized data extraction, formal risk-of-bias assessment, and exploratory feasibility analysis, but before calculation of the definitive pooled estimates, final SWiM synthesis, and GRADE certainty-of-evidence assessment.

### 2.2 Eligibility criteria

Eligibility criteria were defined using two linked Population–Exposure–Comparator–Outcome (PECO) frameworks corresponding to the etiologic and prognostic evidence domains. Both domains included human participants evaluated during acute COVID-19 or after documented SARS-CoV-2 infection. Eligible studies were required to assess glucose regulation and at least one marker of insulin resistance or insulin sensitivity; however, the analytical role of these variables differed between domains. In the etiologic domain, SARS-CoV-2 infection or the transition from acute infection to recovery constituted the exposure or temporal contrast, and glycemic regulation and insulin resistance or insulin sensitivity were evaluated as metabolic outcomes. In the prognostic domain, the exposure was a study-defined acute hyperglycemic phenotype, whereas the assessment of insulin resistance or insulin sensitivity served as a study-level eligibility and contextualization criterion rather than as a component of a participant-level composite exposure.

Studies were eligible if they included participants of any age with laboratory-confirmed or clinically documented SARS-CoV-2 infection during the acute or post-acute phase. No restrictions were applied regarding sex, geographical location, care setting, COVID-19 severity, hospitalization status, or pre-existing diabetes. Studies enrolling mixed populations were included only when data for participants with COVID-19 could be extracted separately.

To establish eligibility for either evidence domain, studies had to report at least one independent measure of glycemic regulation and at least one analytical measure of insulin resistance or insulin sensitivity within the same study population or an analytically linked subgroup. Glycemic assessments included fasting, random, or admission glucose; glycated hemoglobin; oral glucose-tolerance testing; continuous glucose-monitoring variables; or predefined categories such as impaired fasting glucose, impaired glucose tolerance, newly detected diabetes, stress hyperglycemia, acute dysglycemia, or persistent post-COVID hyperglycemia or glucose intolerance.

Insulin resistance or insulin sensitivity could be assessed using a direct physiological method or a recognized surrogate index, including HOMA-IR, HOMA2-IR, HOMA2-%S, QUICKI, the Matsuda index, FIRI, the triglyceride–glucose index, METS-IR, or a comparable validated measure. Studies applying an explicitly defined insulin–glucose or C-peptide–glucose relationship to analytically characterize insulin resistance were also eligible. When an insulin-resistance index intrinsically incorporated glucose, as in the triglyceride–glucose index, an additional independent glycemic variable was required. Measurement of insulin or C-peptide alone, insulin-treatment requirements, or narrative attribution of insulin resistance without an analytical assessment was considered insufficient. Studies evaluating only glycemia or only insulin resistance were excluded.

For the etiologic domain, eligible comparisons included participants with versus without SARS-CoV-2 infection; COVID-19 versus non-COVID acute or critical illness; different COVID-19 severity categories; participants with versus without post-COVID conditions; and within-participant comparisons between acute infection and post-acute follow-up. Studies without an external control group remained eligible when they provided a longitudinal within-participant comparison or another analytical contrast capable of characterizing the development, magnitude, persistence, or resolution of glycemic dysregulation and insulin resistance or insulin sensitivity.

For the prognostic domain, eligible studies compared participants with and without a study-defined acute hyperglycemic phenotype and reported subsequent clinical outcomes. Eligible exposure definitions included newly detected or new-onset hyperglycemia, an elevated stress hyperglycemia ratio, severe hyperglycemia requiring insulin treatment, or investigator-defined acute dysglycemia. Comparator groups comprised participants with normoglycemia, lower relative hyperglycemia, or absence of the study-defined hyperglycemic state. The required insulin-resistance or insulin-sensitivity assessment could have been conducted in the overall study population or an analytically linked subgroup, but it was not required to define the prognostic exposure groups at the participant level.

Outcomes of interest in the etiologic domain included changes in glucose regulation, insulin-resistance or insulin-sensitivity indices, β-cell function, persistent post-COVID metabolic abnormalities, and newly diagnosed or progressive dysglycemia or diabetes. Prognostic outcomes included COVID-19 severity, intensive care admission, invasive or noninvasive ventilatory support, hospitalization duration, and short-term all-cause mortality. Purely descriptive studies without a comparator, temporal contrast, or evaluable association were excluded.

Eligible designs comprised prospective and retrospective cohort studies, case-control studies, analytical cross-sectional studies, and secondary observational or prognostic analyses of clinical trials. Only peer-reviewed full-text reports providing sufficient quantitative information to evaluate the eligible metabolic relationships, temporal changes, or prognostic associations were included. Reviews, meta- analysis, editorials, commentaries, protocols, conference abstracts without a corresponding full report, case reports, purely descriptive case series, preprints, and animal, *in vitro*, or in silico studies were excluded.

### 2.3 Information sources and search strategy

A comprehensive literature search was conducted on June 29, 2026, in PubMed, Scopus, Web of Science, ScienceDirect, and Embase, with coverage extending from the inception of each electronic source to the search date. The search strategy was developed using controlled vocabulary and free-text terms informed by Medical Subject Headings (MeSH; https://www.ncbi.nlm.nih.gov/mesh/) and Health Sciences Descriptors (DeCS; https://decs.bvsalud.org/en/). The three principal concept blocks were combined as follows: (“Insulin Resistance”) AND (“Hyperglycemia”) AND (“COVID-19” OR “SARS-CoV-2”). Synonyms and entry terms corresponding to each concept were combined using the Boolean operator “OR”. The resulting search terms were adapted to the syntax, search fields, and Boolean operators supported by each electronic source while maintaining conceptual equivalence across all searches. No restrictions regarding publication year or language were applied. In addition, the reference lists of the included studies and relevant review articles were manually screened to identify potentially eligible studies not retrieved through the electronic searches. The complete search strategies used for each electronic source, together with the corresponding numbers of records retrieved, are presented in **Supplementary Table 1**.

### 2.4 Study selection and record management

All records retrieved from the electronic searches were exported to Mendeley Reference Manager (https://www.mendeley.com/) and subsequently imported into Rayyan (https://www.rayyan.ai/) for record management and screening. Potential duplicate records were initially identified electronically and then verified using the DOI, normalized title, authorship, and publication year. Duplicate removal was completed before eligibility screening.

Two reviewers independently screened the titles and abstracts of all unique records against the predefined eligibility criteria. Records clearly failing to meet the criteria were excluded at this stage, whereas potentially eligible records and those for which eligibility remained uncertain proceeded to full-text assessment. The corresponding full-text reports were independently evaluated by the same reviewers, and a specific reason for exclusion was recorded for each ineligible report. Reports that could not be obtained after reasonable retrieval efforts were classified as not retrieved rather than as full-text exclusions. Disagreements at either stage were resolved through discussion and consensus; when consensus could not be reached, a third reviewer adjudicated the final decision.

Multiple publications originating from the same underlying study population were identified by comparing authorship, participating centers, country, recruitment period, sample size, participant characteristics, and study procedures. Such publications were linked within a study family and counted as a single independent study. The most complete report was treated as the primary source, while companion publications were retained to obtain additional nonoverlapping outcomes or follow-up data. Overlapping participants or outcomes were not counted more than once in any quantitative synthesis.

### 2.5 Data extraction and outcome definitions

A standardized data-extraction form was developed and pilot-tested on a representative subset of the eligible reports before formal extraction. Two reviewers independently extracted the data from each included publication. Discrepancies were resolved through discussion and consensus, with adjudication by a third reviewer when necessary.

The extracted study-level information comprised the first author, publication year, country, study design, clinical setting, recruitment period, source population, method used to document SARS-CoV-2 infection, phase of COVID-19, sample size, duration of follow-up, and funding or conflict-of-interest information. Participant-level characteristics included age, sex, body mass index, pre-existing diabetes or other metabolic disorders, COVID-19 severity, hospitalization or intensive care status, and exposure to systemic corticosteroids when reported. Information concerning the comparator group, participant selection criteria, missing data, and variables included in adjusted statistical models was also recorded.

For characterization of the metabolic variables, the reviewers extracted the type, definition, unit, threshold, and timing of each glycemic measure and each insulin-resistance or insulin-sensitivity assessment. For prognostic analysis, the variables used to define the exposure and comparator groups were recorded separately from the insulin-resistance or insulin-sensitivity measures used to characterize the study population or an analytically linked subgroup. Glycemic variables included fasting, random, or admission glucose; glycated hemoglobin; oral glucose-tolerance testing; continuous glucose-monitoring variables; and categorical definitions of hyperglycemia, impaired glucose regulation, or newly diagnosed diabetes. Insulin-resistance or insulin-sensitivity measures included direct physiological assessments and recognized surrogate indices, such as HOMA-IR, HOMA2-IR, HOMA2-%S, QUICKI, the Matsuda index, FIRI, the triglyceride–glucose index, and METS-IR, together with explicitly defined insulin–glucose or C-peptide–glucose relationships. The formula and cutoff used for each index were retained as reported by the original investigators.

Extracted outcomes were organized into two related but analytically distinct evidence domains. The etiologic domain comprised the relationships between SARS-CoV-2 infection or clinical phase and the magnitude, temporal evolution, or persistence of glycemic dysregulation and insulin resistance or insulin sensitivity during acute and post-acute COVID-19. The prognostic domain comprised associations between study-defined acute hyperglycemic phenotypes and clinically relevant outcomes, including disease severity, intensive care admission, ventilatory support, hospitalization duration, and mortality, in studies that also assessed insulin resistance or insulin sensitivity. Post-COVID conditions, persistent glycemic abnormalities, and newly diagnosed diabetes were retained as etiologic or longitudinal metabolic outcomes according to the analytical role assigned by each study. The exact timing of each measurement was extracted, and repeated assessments were retained separately to characterize longitudinal changes.

For continuous outcomes, group means and standard deviations, medians and interquartile ranges, change scores, and corresponding sample sizes were extracted as available. For binary outcomes, event counts and group totals were recorded. Reported odds ratios, risk ratios, hazard ratios, regression coefficients, and their corresponding 95% confidence intervals or standard errors were extracted for association analyses. When several statistical models were reported for the same outcome, the most comprehensively adjusted estimate was prioritized, and the covariates included in that model were documented.

Publications originating from the same underlying cohort were consolidated at the study-family level. The report containing the most complete methodological and outcome information was designated as the primary source, while companion publications contributed only unique outcomes, additional follow-up assessments, or nonoverlapping subgroup data. No participant group, outcome, or follow-up interval was entered more than once in the same quantitative synthesis.

For studies included in the prognostic meta- analysis, we additionally extracted the exact exposure and comparator definitions, glycemic thresholds, timing of exposure ascertainment, insulin-resistance or insulin-sensitivity measures, eligibility or classification according to pre-existing diabetes, corticosteroid exposure, outcome definitions, and the number of events and participants in each comparison group.

### 2.6 Risk-of-bias assessment

Risk of bias was assessed at the level of each eligible result, with the appraisal tool selected according to the target estimand and study design. Non-randomized follow-up studies estimating the etiological effect of SARS-CoV-2 exposure were evaluated using the Risk Of Bias In Non-randomized Studies of Exposures (ROBINS-E) tool (14). Observational studies that did not meet the design requirements of ROBINS-E were assessed using the revised Joanna Briggs Institute critical appraisal tools appropriate for cohort, analytical cross-sectional (15), or case-control studies (16). Studies examining glycemic, insulin-resistance, or inflammatory variables as prognostic factors for subsequent clinical or metabolic outcomes were evaluated using the Quality In Prognosis Studies (QUIPS) tool (17). For secondary analysis derived from randomized controlled trials, the revised Cochrane Risk of Bias tool (RoB 2) was applied to the effect of assignment to the randomized intervention (18).

Potential confounding factors were defined before the assessments. For etiological results, the minimum confounder set comprised age, sex, adiposity, pre-existing diabetes or baseline glycemic status, and cardiometabolic comorbidities; COVID-19 severity, glucocorticoid exposure, relevant treatments, and the timing of outcome assessment were additionally considered when applicable. For prognostic results, the assessment considered age, sex, adiposity, previous diabetes or baseline HbA1c, acute disease severity or oxygenation status, glucocorticoid administration, relevant comorbidities or organ dysfunction, and concurrent treatments. When multiple publications originated from the same underlying population, shared study-level domains were assessed once, whereas outcome- or analysis-specific domains were evaluated separately to prevent duplicate assessment.

Two reviewers independently completed the signaling questions and domain-level judgments for each eligible result. Disagreements were resolved through discussion and consensus, with consultation of a third reviewer when necessary. Judgments followed the categories and decision algorithms specified by each instrument. ROBINS-E assessments considered seven bias domains and generated domain-level and overall judgments for each etiological result (14). QUIPS assessments covered study participation, study attrition, prognostic-factor measurement, outcome measurement, adjustment for confounding, and statistical analysis and reporting (17). RoB 2 assessments addressed the randomization process, deviations from intended interventions, missing outcome data, outcome measurement, and selection of the reported result (18). For the JBI tools, responses were recorded as “yes,” “no,” “unclear,” or “not applicable” and interpreted at the item and bias-domain levels without calculating an aggregate numerical quality score (15, 16). Risk-of-bias judgments were not used as eligibility criteria but were incorporated into the interpretation of the synthesized evidence. Detailed assessments and graphical summaries were generated separately for each instrument because their domains and judgment categories are not directly interchangeable.

### 2.7 Data synthesis and statistical analysis

Reports derived from the same or partially overlapping population were grouped as a single study family. For each outcome and assessment time, the most complete report was selected. Companion publications contributed only non-overlapping outcomes or follow-up periods, thereby avoiding duplicate participant inclusion.

Evidence synthesis was structured according to the etiologic or prognostic role of each study. For etiologic evidence, SARS-CoV-2 infection or clinical phase was considered the exposure or temporal contrast, and the evaluated outcomes included the development, magnitude, persistence, or resolution of insulin resistance, impaired insulin sensitivity, glycemic dysregulation, or diabetes. For the quantitative synthesis of prognostic outcomes, the exposure was operationalized as a study-defined acute hyperglycemic phenotype, including new-onset hyperglycemia, an elevated stress hyperglycemia ratio, severe hyperglycemia, or investigator-defined acute dysglycemia. Hyperglycemia, rather than a participant-level combination of hyperglycemia and insulin resistance, defined the prognostic exposure and comparator groups. Assessment of at least one concurrent marker of insulin resistance or insulin sensitivity was required for study eligibility and metabolic contextualization but was not assumed to form part of the prognostic exposure.

Quantitative synthesis was performed when at least three independent studies reported the same prognostic outcome and their exposure definitions represented an acute hyperglycemic state during the index hospitalization. Clinical comparability required: (i) hospitalized patients with acute COVID-19; (ii) a dichotomous comparison between a study-defined hyperglycemic or dysglycemic group and a normoglycemic or lower-glycemia reference group; (iii) ascertainment of the glycemic exposure during the index hospitalization; (iv) compatible definitions and assessment periods for the clinical outcome; and (v) sufficient event data to reconstruct a 2 × 2 table. New-onset hyperglycemia, an elevated stress hyperglycemia ratio, severe hyperglycemia requiring initiation of insulin therapy, and dysglycemia identified from repeated inpatient glucose profiles were regarded as clinically related manifestations of a broader acute hyperglycemic exposure class, but not as interchangeable diagnostic thresholds. Consequently, the pooled RRs were interpreted as average associations across study-specific definitions rather than as the effect associated with a single glucose threshold or uniform diagnostic entity.

Eligible outcomes included all-cause mortality, invasive mechanical ventilation or endotracheal intubation, intensive care unit admission, disease severity, length of hospital stay, and hospital readmission. Outcome definitions and assessment periods were evaluated for clinical comparability before pooling. Short-term mortality encompassed death during the index hospitalization or within 60 days, whereas invasive mechanical ventilation included intubation during the index hospitalization. For dichotomous outcomes, unadjusted risk ratios (RRs) and 95% confidence intervals (CIs) were calculated from reported or reconstructed 2 × 2 tables. This common event-count estimand was selected because the required data were available for every study contributing to each pooled outcome, whereas compatible adjusted estimates for the same exposure–outcome contrasts were available from only one study. Adjusted associations were extracted and synthesized narratively when available but were not combined with unadjusted RRs because they differed in effect measure, exposure parameterization, covariate selection, and model specification. Adjusted odds ratios were not treated as interchangeable with RRs, particularly because the outcomes were not rare. Consequently, the pooled RRs quantify total, unadjusted prognostic associations and should not be interpreted as effects independent of confounding.

Pooled estimates were calculated using inverse-variance random-effects models. Between-study variance was estimated using restricted maximum likelihood, and 95% CIs were calculated using the modified Hartung–Knapp method. Statistical heterogeneity was evaluated using Cochran’s Q and quantified using τ^2^ and I^2^. Prediction intervals were calculated when statistically estimable. A continuity correction of 0.5 was applied only to studies containing a zero cell, whereas studies with no events in either comparison group were excluded from the corresponding RR analysis. All tests were two-sided, with p<0.05 considered statistically significant.

Sensitivity analysis included leave-one-out analysis, exclusion of studies with a high or serious risk of bias, and the addition or exclusion of studies that defined the acute hyperglycemic exposure using the maximum glucose value recorded at any time during hospitalization. Definitions based exclusively on peak in-hospital glucose were considered less temporally comparable with admission-based or prospectively characterized inpatient glycemic phenotypes because the peak measurement could occur after clinical deterioration or after exposure to corticosteroids, nutritional support, or intensive-care treatment. These studies were therefore excluded from the primary analysis and evaluated only in sensitivity analysis. Potential sources of heterogeneity, including pre-existing diabetes, exposure definition and timing, corticosteroid exposure, disease severity, and comparator selection, were examined descriptively. Formal subgroup analysis were planned only when sufficient comparable data were available. Meta-regression, funnel plots, and statistical tests for small-study effects were planned only when at least 10 independent studies contributed to an outcome.

Outcomes and effect estimates that could not be combined quantitatively were synthesized according to the Synthesis Without Meta-analysis reporting guideline (19). The synthesis was organized according to evidence function. Etiological evidence included infection-associated insulin resistance and glucose dysregulation, longitudinal or persistent post-COVID metabolic abnormalities, incident or progressive glucose abnormalities or diabetes, and mechanistic findings reported within eligible clinical studies. Non-combinable prognostic evidence comprised outcomes or estimates not eligible for pooling, including data on mortality, invasive mechanical ventilation, intensive care unit admission, disease severity, length of hospital stay, and readmission. Within each group, findings were compared according to population, comparator, clinical phase, metabolic measure, effect magnitude and direction, precision, adjustment for confounding, and risk of bias. Conclusions were based on the consistency and methodological credibility of the evidence rather than on statistical-significance vote counting. Analyses were performed using R version 4.5.2 (https://cran.r-project.org/manuals.html), with the meta version 8.5-0 (20) and metafor version 5.0–1 packages (21).

### 2.8 Assessment of the certainty of evidence

The certainty of evidence was assessed independently by two reviewers using the Grading of Recommendations Assessment, Development and Evaluation (GRADE) approach. Disagreements were resolved by consensus, with consultation of a third reviewer when necessary. GRADE assessments were conducted separately for each clinically relevant outcome and according to its etiological or prognostic function. Observational evidence addressing prognostic factors or exposures initially received a high-certainty rating, consistent with GRADE guidance for prognostic and exposure questions (22, 23).

For the prognostic component, certainty was assessed for short-term all-cause mortality and invasive mechanical ventilation. The target of assessment was confidence in the association between the acute hyperglycemic phenotype and each clinical outcome rather than certainty in a causal effect. For the etiological component, certainty was assessed for infection-associated insulin resistance or glycemic dysregulation, persistent post-COVID metabolic abnormalities, and incident or progressive dysglycemia or diabetes. Other non-combinable prognostic outcomes were assessed when a coherent body of evidence was available. Mechanistic biomarkers were not assigned an independent GRADE rating because they were used to support biological plausibility rather than represent patient-important outcomes.

Certainty could be downgraded for risk of bias, inconsistency, indirectness, imprecision, or publication bias. The GRADE risk-of-bias domain was assessed at the outcome and body-of-evidence level rather than by converting the categories of the different appraisal tools into a common numerical score. For each GRADE outcome, every contributing result was linked to its corresponding ROBINS-E, QUIPS, JBI, or RoB 2 assessment. The original categories of each instrument were retained: low risk, some concerns, high risk, or very high risk for ROBINS-E; low, moderate, or high risk for QUIPS; and low risk, some concerns, or high risk for RoB 2. JBI responses were retained as “yes,” “no,” “unclear,” or “not applicable” and were not summed or converted into an automatic overall rating. Across instruments, reviewers considered conceptually corresponding sources of bias, including confounding, participant selection or study participation, missing data or attrition, exposure or prognostic-factor measurement, outcome measurement, selective reporting, and, when applicable, the randomization process and deviations from intended interventions. The body of evidence was downgraded one level for serious risk of bias when the contributing studies showed recurrent concerns in outcome-relevant domains or when an important proportion of the evidence came from studies with high overall risk of bias. Decisions considered each study’s contribution to the outcome and effect estimate rather than study counts or a mechanical crosswalk between instruments. Inconsistency was evaluated from the direction and magnitude of study-level effects and, for pooled outcomes, from τ^2^, I^2^, and the overlap of confidence intervals. Indirectness was judged according to differences in population, exposure definition, comparator, outcome measurement, and assessment time relative to the review question. Imprecision was evaluated using confidence intervals, sample size, number of events, and whether the evidence supported materially different interpretations. Publication bias was assessed from the comprehensiveness of the search, selective outcome reporting, and the distribution of study sizes; statistical tests for small-study effects were considered only when at least 10 studies were available.

For outcomes synthesized without meta-analysis, the same GRADE domains were applied to the body of evidence using the direction, magnitude, precision, and consistency of the study-level findings, without requiring a single pooled effect estimate (24). Final certainty was categorized as high, moderate, low, or very low, and all downgrading decisions were documented using explanatory footnotes. Evidence profiles and Summary of Findings tables were prepared using GRADEpro GDT (https://www.gradepro.org).

## 3 Results

### 3.1 Study selection

The electronic searches identified 2763 records: 94 from PubMed, 293 from Scopus, 126 from Web of Science, 1910 from ScienceDirect, and 340 from Embase. After the removal of 472 duplicate records, 2291 unique records underwent title and abstract screening. Of these, 2216 were excluded because they did not meet the predefined eligibility criteria, leaving 75 reports for retrieval. Manual screening of the reference lists of included studies and relevant reviews identified two additional potentially eligible reports, resulting in 77 reports sought for full-text assessment.

Two reports could not be retrieved despite reasonable efforts; consequently, 75 full-text reports were assessed for eligibility. Of these, 52 were excluded: 39 did not provide a valid insulin-resistance or insulin-sensitivity assessment alongside an eligible glycemic assessment; seven used insulin administration or treatment requirements as a proxy for insulin resistance; three assessed insulin resistance without an independent glycemic component; two provided only preclinical or mechanistic evidence without eligible concurrent clinical assessments of glycemic regulation and insulin resistance or insulin sensitivity; and one was an ineligible nonprimary publication. Ultimately, 23 publications fulfilled all eligibility criteria and were included in the systematic review. Because two study families were represented by multiple companion publications, these 23 reports corresponded to 20 independent studies. The complete study-selection process is presented in the PRISMA 2020 flow diagram (**Figure 1**).

**Figure 1:**
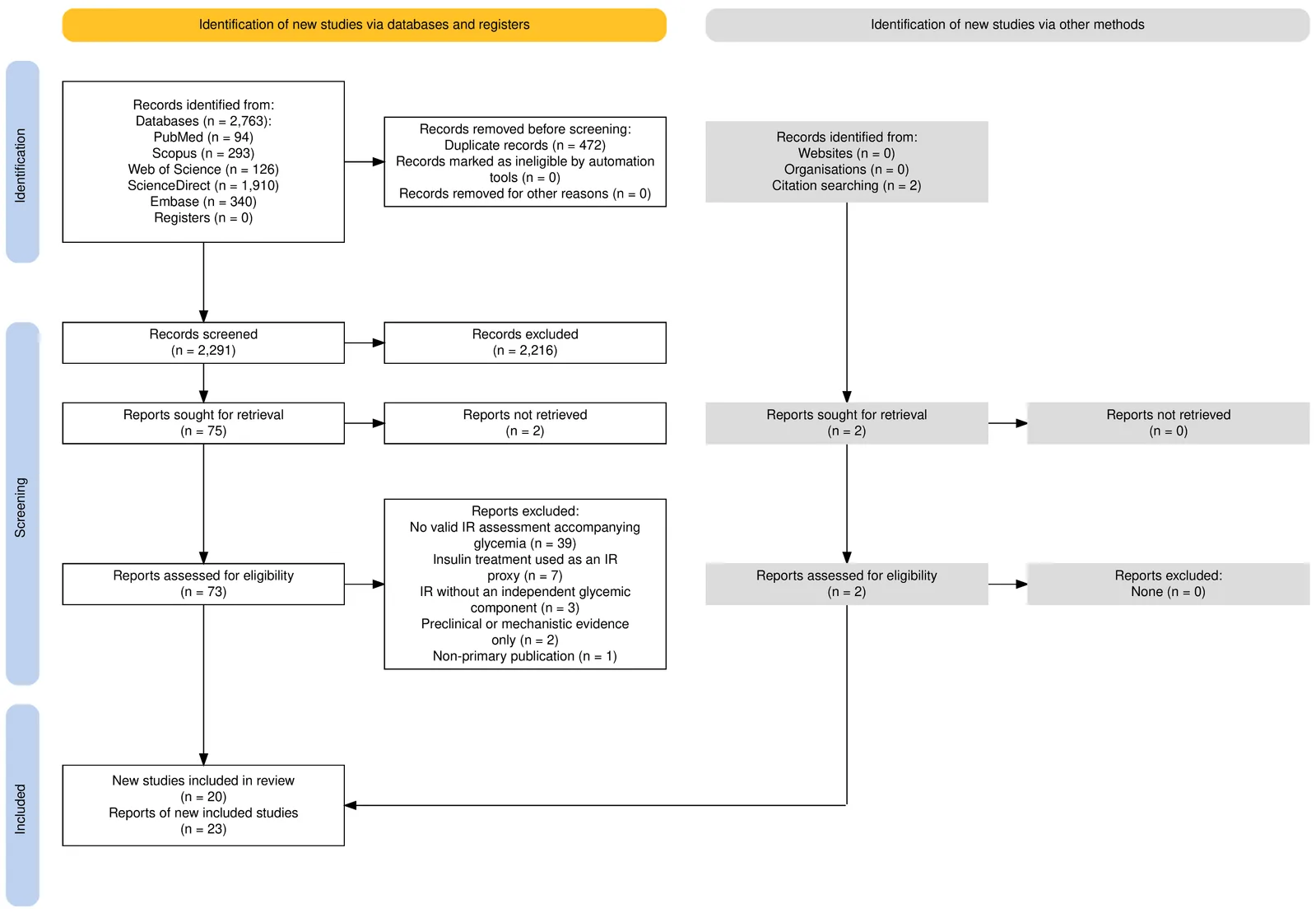
PRISMA 2020 flow diagram of the study-selection process. The diagram summarizes the identification of records through five electronic databases and citation searching, duplicate removal, title and abstract screening, full-text eligibility assessment, reasons for exclusion, and final inclusion of 23 publications representing 20 independent studies in the systematic review.

### 3.2 Characteristics of the included studies

The systematic review included 23 publications (25–47) representing 20 independent studies or study families (**Table 1**). The two publications by Calcaterra et al. reported different follow-up stages of the same pediatric MIS-C cohort and were therefore treated as a single study family (28, 35). Similarly, the three publications by Tsvetkova et al. were considered a partially overlapping immunometabolic research program and were consolidated at the study-family level to prevent double-counting (44–46). Publication years ranged from 2021 to 2026 (25–47). The evidence originated from 14 countries or territories. Studies were conducted in China (25, 29), the United States (26), India (27, 40), Italy (28, 31, 32, 35), Greece (30, 37), Mexico (33), Ghana (34), and the Czech Republic (36). Additional studies were conducted in Romania (38), the Netherlands (39), Hong Kong SAR, China (41), Iraq (42, 43), Bulgaria (44–46), and Croatia (47).

**Table 1:** General characteristics of the included studies.

Study	Country	Design and setting	COVID-19 phase and follow-up	Study population	Sample size	Main comparator or analytical contrast
Chen et al., 2021 (25)	China	Prospective longitudinal cohort; hospital and outpatient follow-up	Acute infection; 3 and 6 months	Adults with confirmed COVID-19 and no previous diabetes	64	Within-person metabolic changes from admission to follow-up
Reiterer et al., 2021 (26)	United States	Multicenter hospital cohort with mechanistic subcohorts	Acute hospitalization	Adults hospitalized with COVID-19, with and without pre-existing diabetes	3,854	ARDS versus no ARDS; hyperglycemia versus no hyperglycemia; selected critical-care controls
Goyal et al., 2021 (27)	India	Established longitudinal community cohort	Pre-COVID and peri-COVID assessments; mean interval of 21.5 months	Adults with longitudinal glycemic and cardiometabolic measurements	352	SARS-CoV-2-infected versus non-infected participants; within-person changes
Calcaterra et al., 2021 and 2023 (28, 35)	Italy	Prospective pediatric case series with longitudinal follow-up	Acute MIS-C; 6 and 12 months	Children and adolescents with MIS-C related to SARS-CoV-2	38	Within-person changes; no external comparison group
He et al., 2021 (29)	China	Comparative cohort with metabolomic and proteomic substudies	Acute infection and recovery	Patients with COVID-19 without previous metabolic disease and healthy controls	154	COVID-19 versus healthy controls; non-severe versus severe disease
Ilias et al., 2021 (30)	Greece	Hospital cohort; COVID-19 ward and ICU	Acute hospitalization	Adults with mild-to-critical COVID-19, with and without diabetes	157	Ward versus ICU; diabetes versus no diabetes; survivors versus non-survivors
Montefusco et al., 2021 (31)	Italy	Observational hospital cohort with metabolic substudies	Acute infection and approximately 2-month follow-up	Hospitalized adults with diabetes, newly detected hyperglycemia, or normoglycemia	551	Pre-existing diabetes versus new hyperglycemia versus normoglycemia
Da Porto et al., 2022 (32)	Italy	Prospective hospital cohort	Acute hospitalization; 60-day outcomes	Adults hospitalized with COVID-19, including participants with diabetes	150	Stress hyperglycemia ratio >1.14 versus ≤1.14
Soto et al., 2022 (33)	Mexico	Comparative analytical cohort; referral center	Acute moderate or severe disease	Patients with COVID-19 and healthy controls	86	COVID-19 versus healthy controls
Adatsi et al., 2023 (34)	Ghana	Post-acute comparative study; community and laboratory assessment	Approximately 6–8 months after infection	Recovered adults and individuals without previous SARS-CoV-2 infection	226	Recovered COVID-19 participants versus non-infected controls
Gojda et al., 2023 (36)	Czech Republic	Prospective hospital cohort with follow-up	Acute severe disease; 3 and 6 months	Survivors of severe COVID-19 without previously known diabetes	26	Acute hyperglycemia versus normoglycemia; within-person changes
Liontos et al., 2023 (37)	Greece	Retrospective hospital cohort	Acute hospitalization	Adults with diabetes, obesity, both conditions, or neither condition	1,458	Comparisons across diabetes, obesity, and metabolically unaffected strata
Andor et al., 2024 (38)	Romania	Retrospective longitudinal cohort; hospital and follow-up	Hospital discharge; 4 and 12 months	Patients with moderate-to-severe COVID-19 and no previous diabetes	135	Within-person metabolic changes and predictors of subsequent glycemia
Duijvelaar et al., 2024 (39)	Netherlands	Secondary biomarker analysis of a multicenter randomized controlled trial conducted in 13 hospitals	Acute severe disease; hospital course and days 2–3 biomarker assessments	Adults hospitalized with severe COVID-19	385	Imatinib versus placebo; severe hyperglycemia versus no severe hyperglycemia
Khan et al., 2024 (40)	India	Observational hospital cohort	Acute moderate-to-critical disease	Adults with COVID-19 and no previous diabetes	126	Survivors versus non-survivors; lower versus higher baseline insulin resistance
Lui et al., 2024 (41)	Hong Kong SAR, China	Prospective cohort with matched controls; hospital and community follow-up	Approximately 6 weeks and 1 year after acute infection	COVID-19 survivors with dysglycemia during acute infection and matched controls	102	COVID-19 survivors versus controls at baseline; within-person changes at 1 year
Al Masoodi et al., 2025 (42)	Iraq	Case–control study; community and laboratory assessment	Long COVID and post-recovery	Individuals with long COVID and recovered participants without long COVID; diabetes excluded	90	Long COVID versus recovered participants without long COVID
Ahmed et al., 2025 (43)	Iraq	Comparative cross-sectional study; hospital and outpatient assessment	Post-hospitalization during the Omicron period	Adults with T2DM and COVID-19, T2DM without COVID-19, and healthy controls	150	T2DM with COVID-19 versus T2DM without COVID-19 versus healthy controls
Tsvetkova et al., 2025–2026 (44–46)	Bulgaria	Comparative immunometabolic research program with partially overlapping reports	Active COVID-19 and post-COVID metabolic disease	Participants with active COVID-19, newly developed post-COVID metabolic disorders, or classic metabolic syndrome	168	Post-COVID metabolic disorders versus classic metabolic syndrome; subgroup comparisons
Grubišić et al., 2026 (47)	Croatia	Prospective hospital cohort with longitudinal follow-up	Acute hospitalization; 3 and 6 months	Adults hospitalized with COVID-19 and no previous diabetes	144	Normoglycemia versus stress hyperglycemia versus new-onset diabetes

The 23 included publications represented 20 independent studies or study families. Sample size refers to the largest non-overlapping analytical cohort used for general study characterization; outcome-specific analysis may have used smaller denominators. The paired reports by Calcaterra et al. (28, 35) and the partially overlapping reports by Tsvetkova et al. (44–46) were consolidated at the study-family level. ARDS, acute respiratory distress syndrome; COVID-19, coronavirus disease 2019; ICU, intensive care unit; MIS-C, multisystem inflammatory syndrome in children; SARS-CoV-2, severe acute respiratory syndrome coronavirus 2; T2DM, type 2 diabetes mellitus.

The included studies used heterogeneous but complementary methodological designs. Prospective longitudinal or follow-up cohorts evaluated glycemic and insulin-related changes during acute infection and subsequent recovery (25, 27, 28, 35, 36, 41, 47). Da Porto et al. conducted a prospective hospital cohort with assessment of 60-day clinical outcomes (32), whereas Montefusco et al. combined an acute hospital-based metabolic assessment with an approximately 2-month follow-up evaluation (31). Retrospective designs were used to examine metabolic alterations during hospitalization and after discharge (37, 38), while other observational hospital cohorts evaluated acute glycemic dysregulation, insulin resistance, disease severity, or mortality (26, 30, 40). Comparative studies incorporated healthy or non-infected control groups and, in some cases, metabolomic, proteomic, or oxidative-stress assessments (29, 33, 34). The remaining evidence included a secondary biomarker analysis of a multicenter randomized controlled trial (39), a case–control study of long COVID (42), a comparative cross-sectional study involving patients with T2DM (43), and a series of partially overlapping comparative immunometabolic investigations (44–46).

Sample sizes varied substantially according to study design and setting, ranging from 26 participants in the prospective cohort reported by Gojda et al. (36) to 3854 hospitalized patients in the multicenter cohort reported by Reiterer et al. (26). Most investigations enrolled adult participants across hospital, intensive-care, outpatient, community, or post-COVID follow-up settings. The only pediatric study family evaluated children and adolescents with MIS-C during the acute phase and at 6- and 12-month follow-up (28, 35). Participants without previously diagnosed diabetes or metabolic disease were specifically selected in several cohorts (25, 29, 36, 38, 40, 47), while diabetes was excluded from the long-COVID case-control study conducted by Al Masoodi et al. (42). Other investigations included participants with and without pre-existing diabetes or explicitly stratified the population according to diabetes, newly detected hyperglycemia, obesity, or normoglycemia (26, 30, 31, 37, 43). Lui et al. specifically followed COVID-19 survivors who had developed dysglycemia during the acute infection and compared them with matched controls (41).

The timing of metabolic assessment encompassed both acute and post-acute COVID-19. Acute hospitalized populations were evaluated in relation to hyperglycemia, insulin resistance, critical illness, or clinical outcomes (26, 30, 33, 37, 39). Several studies extended the assessment beyond hospitalization. Metabolic outcomes were evaluated at 3 and 6 months in the cohorts reported by Chen et al. (25), Gojda et al. (36), and Grubišić et al. (47); at 6 and 12 months in the pediatric MIS-C study family (28, 35); and at 4 and 12 months after hospital discharge in the cohort reported by Andor et al. (38). Montefusco et al. performed an approximately 2-month post-acute assessment (31), whereas Lui et al. evaluated survivors at approximately 6 weeks and again at 1 year (41). Goyal et al. compared pre-COVID and peri-COVID cardiometabolic measurements obtained over a mean interval of 21.5 months (27). Exclusively post-acute or recovery-based populations were examined approximately 6–8 months after infection in Ghana (34), in individuals with and without long COVID in Iraq (42), after COVID-19 hospitalization among patients with T2DM (43), and among individuals with newly developed post-COVID metabolic disorders in Bulgaria (44–46).

The analytical comparisons reflected the etiologic and prognostic dimensions of the review question. Comparisons between participants with previous SARS-CoV-2 infection and healthy, non-infected, or matched controls were performed in studies from India, China, Mexico, Ghana, and Hong Kong SAR (27, 29, 33, 34, 41). Within-person metabolic changes across acute infection and follow-up were evaluated in longitudinal cohorts from China, India, Italy, the Czech Republic, Romania, Hong Kong SAR, and Croatia (25, 27, 28, 35, 36, 38, 41, 47). Acute prognostic contrasts included ARDS and hyperglycemia status (26), ward versus ICU admission and survival status (30), stress hyperglycemia categories (32), diabetes and obesity strata (37), severe hyperglycemia and randomized treatment allocation (39), baseline insulin resistance and hospital mortality (40), and acute glycemic phenotypes associated with disease severity (47). Additional studies compared pre-existing diabetes, newly detected hyperglycemia, and normoglycemia (31); T2DM with and without previous COVID-19 (43); or post-COVID metabolic disorders and classic metabolic syndrome (44–46).

Collectively, these studies provided a clinically and temporally diverse evidence base encompassing acute glycometabolic disruption, inflammation-associated insulin resistance, hyperglycemia, β-cell dysfunction, persistent post-COVID metabolic abnormalities, and progression toward newly developed metabolic disease. This heterogeneity supported the prespecified organization of the evidence according to acute versus post-acute disease phase and etiologic versus prognostic analytical contrasts.

### 3.3 Synthesis without meta-analysis of etiological evidence

Etiological findings that could not be combined quantitatively were synthesized without meta-analysis and are presented according to the prespecified metabolic domains summarized in **Table 2**. Evidence mapping identified hyperglycemia and glucose dysregulation as the most consistently evaluated metabolic domain, constituting a primary domain in 18 studies and a secondary domain in the remaining two studies. Acute glycometabolic disruption was classified as primary in 14 studies and secondary in three, while inflammation-driven insulin resistance was primary in 13 studies and secondary in six. Evidence concerning β-cell dysfunction was less uniform, being primary in eight studies and secondary in nine. Persistent post-COVID metabolic abnormalities were directly evaluated in 12 studies, whereas progression toward new-onset diabetes constituted a primary domain in only four studies and a secondary domain in nine. These classifications indicate the centrality of each domain within the individual study rather than the direction or statistical significance of its findings.

**Table 2:** Evidence mapping matrix of glycemic dysregulation and insulin resistance across acute and post-acute COVID-19.

Study	D1	D2	D3	D4	D5	D6	Evidence synthesis
Chen et al., 2021 (25)	S	S	P	P	P	S	Reduced C-peptide during acute infection was followed by recovery of secretion, but TyG remained elevated at 3 and 6 months, indicating persistent insulin resistance without overt diabetes.
Reiterer et al., 2021 (26)	P	P	P	S	NE/NC	NE/NC	Acute COVID-19-associated hyperglycemia was driven predominantly by insulin resistance linked to adipose-tissue infection, inflammatory dysfunction, and reduced adiponectin rather than β-cell failure.
Goyal et al., 2021 (27)	NE/NC	NE/NC	P	P	P	P	Predominantly mild or asymptomatic infection was associated with BMI progression but not with significantly greater glycemic deterioration, insulin resistance, or β-cell dysfunction than in non-infected participants.
Calcaterra et al., 2021 and 2023 (28, 35)	P	P	P	S	P	S	MIS-C produced marked acute insulin resistance and glycemic fluctuation; abnormalities improved but persisted in a subset at 6 and 12 months without progression to overt diabetes.
He et al., 2021 (29)	P	P	P	S	P	S	SARS-CoV-2 induced new-onset insulin resistance and metabolic dysregulation through altered secreted metabolic factors; insulin resistance persisted into recovery and was not explained by insulin deficiency.
Ilias et al., 2021 (30)	P	S	P	P	NE/NC	S	Acute hyperglycemia increased with clinical severity, while critically ill patients exhibited both reduced insulin sensitivity and compromised insulin secretion.
Montefusco et al., 2021 (31)	P	P	P	P	P	S	Acute new hyperglycemia and insulin resistance coexisted with β-cell hyperstimulation and abnormal cytokine profiles, with glycometabolic abnormalities persisting after recovery.
Da Porto et al., 2022 (32)	P	P	P	NE/NC	NE/NC	NE/NC	Stress hyperglycemia was associated with cytokine release, insulin resistance, and worse clinical outcomes during acute hospitalization.
Soto et al., 2022 (33)	P	P	P	S	NE/NC	S	Hyperglycemia, hyperinsulinemia, and HOMA-IR were accompanied by oxidative and nitrosative stress, linking glucose-insulin disruption with loss of redox homeostasis.
Adatsi et al., 2023 (34)	NE/NC	S	S	P	P	S	Recovered participants exhibited persistent insulin resistance and reduced β-cell function, indicating sustained post-COVID diabetogenic vulnerability.
Gojda et al., 2023 (36)	P	S	P	P	P	S	Acute insulin resistance resolved during follow-up, whereas persistent hyperglycemia was predominantly associated with an insufficient insulin response and β-cell dysfunction.
Liontos et al., 2023 (37)	P	P	P	NE/NC	NE/NC	NE/NC	Inflammatory markers, insulin-resistance indices, and hyperglycemia jointly contributed to prolonged hospitalization and adverse outcomes across diabetes and obesity strata.
Andor et al., 2024 (38)	S	P	P	NE/NC	P	S	Glycemia, HOMA-IR, and high-sensitivity CRP at discharge predicted glycemic levels at 4 and 12 months, connecting acute immunometabolic disruption with persistent glycemic abnormalities.
Duijvelaar et al., 2024 (39)	P	P	P	S	NE/NC	NE/NC	Imatinib reduced severe hyperglycemia and inflammatory JNK/IL-6 signaling while increasing adiponectin, without altering insulin production, supporting restoration of peripheral glucose uptake.
Khan et al., 2024 (40)	P	S	P	S	NE/NC	NE/NC	Baseline HOMA-IR and hyperglycemia predicted hospital mortality with moderate discrimination among patients without previous diabetes.
Lui et al., 2024 (41)	S	S	P	P	P	P	Insulin resistance characterized survivors with acute dysglycemia, and 23.8% experienced glycemic progression at 1 year despite stable C-peptide levels.
Al Masoodi et al., 2025 (42)	NE/NC	P	S	S	P	NE/NC	Long COVID was characterized by persistent insulin resistance and galanin-system activation, with CRP and insulin/glucose measures contributing to diagnostic discrimination.
Ahmed et al., 2025 (43)	P	P	P	S	S	NE/NC	Patients with T2DM and COVID-19 exhibited higher fasting and random glucose, fasting insulin, HOMA-IR, and CRP than patients with T2DM without COVID-19, supporting inflammatory aggravation of established metabolic dysfunction.
Tsvetkova et al., 2025-2026 (44–46)	P	P	P	P	P	P	The partially overlapping reports linked immune imbalance, oxidative stress, insulin resistance, and β-cell dysfunction with newly developed post-COVID carbohydrate disorders and persistent immunometabolic activation.
Grubišić et al., 2026 (47)	P	P	P	S	P	P	Acute hyperglycemia and cytokine activation identified severe disease, while higher HOMA-IR distinguished persistent newly diagnosed diabetes from transient stress hyperglycemia at 3 and 6 months.

P (Primary) indicates that the domain was directly evaluated and central to the study, regardless of whether the result was positive or negative. S (Secondary) indicates indirect, supportive, ancillary, or incomplete evidence. NE/NC (not evaluated or not central) indicates that the domain was not assessed or was not relevant to the study aims. D1, acute glycometabolic disruption; D2, inflammation-driven insulin resistance; D3, hyperglycemia and glucose dysregulation; D4, β-cell dysfunction and impaired insulin secretion; D5, persistent post-COVID metabolic abnormalities; D6, progression toward new-onset diabetes. The paired reports by Calcaterra et al. (28, 35) and the partially overlapping reports by Tsvetkova et al. (44–46) were consolidated at the study-family level to avoid double-counting. BMI, body mass index; COVID-19, coronavirus disease 2019; CRP, C-reactive protein; HOMA-IR, Homeostatic Model Assessment of Insulin Resistance; IL-6, interleukin 6; JNK, c-Jun N-terminal kinase; MIS-C, multisystem inflammatory syndrome in children; SARS-CoV-2, severe acute respiratory syndrome coronavirus 2; T2DM, type 2 diabetes mellitus; TyG, triglyceride-glucose index.

Acute COVID-19 was predominantly characterized by the concurrence of hyperglycemia and impaired insulin sensitivity. In critically ill adults, insulin resistance rather than insulin deficiency was identified as the principal mechanism underlying hyperglycemia and was linked to SARS-CoV-2 infectivity of adipose tissue and reduced circulating adiponectin (26). New-onset insulin resistance accompanied by glucose and lipid dysregulation was also documented during infection and persisted after viral clearance (29). Other hospitalized cohorts demonstrated that critical illness could involve both reduced insulin sensitivity and compromised insulin secretion (30), while acute hyperglycemia and insulin resistance coexisted with β-cell hyperstimulation and abnormal cytokine profiles (31). Acute insulin resistance was also observed in children with MIS-C, together with pathological HOMA-IR, TyG values, and glycemic fluctuations despite the absence of obesity or previous diabetes (28, 35).

Associations with inflammatory pathways were supported by several complementary lines of evidence. Stress hyperglycemia was associated with cytokine release and adverse clinical outcomes among hospitalized patients (32), whereas hyperglycemia, hyperinsulinemia, and HOMA-IR were accompanied by oxidative and nitrosative stress and depletion of antioxidant defenses (33). Inflammatory markers, TyG-derived insulin-resistance indices, and hyperglycemia were associated with prolonged hospitalization or mortality across diabetes and obesity strata (37). Imatinib reduced the incidence of severe hyperglycemia while decreasing IL-6 and JNK signaling and increasing adiponectin, supporting an inflammation-mediated impairment of peripheral glucose uptake (39). At discharge, glycemia, HOMA-IR, and high-sensitivity C-reactive protein predicted subsequent glycemia at 4 and 12 months (38). In long COVID, C-reactive protein, insulin/glucose measures, and activation of the galanin system jointly discriminated affected participants from recovered controls (42). The Bulgarian study family further linked persistent cytokine imbalance, oxidative stress, insulin resistance, and β-cell dysfunction with newly developed post-COVID carbohydrate disorders (44–46). Admission cytokine activation was also associated with acute hyperglycemia and disease severity, although inflammatory burden alone did not distinguish transient stress hyperglycemia from persistent newly diagnosed diabetes (47).

Evidence concerning β-cell function revealed important mechanistic heterogeneity. Reduced C-peptide during acute infection was followed by recovery of insulin secretion, while insulin-resistance indices remained elevated during follow-up (25). Predominantly mild or asymptomatic infection was not associated with greater deterioration of oral disposition index, insulin sensitivity, or glycemic status than that observed in non-infected participants (27). By contrast, compromised insulin secretion was observed among critically ill patients (30), and persistent hyperglycemia after severe COVID-19 was attributed primarily to an insufficient insulin response after acute insulin resistance had resolved (36). Recovered participants from Ghana exhibited persistent insulin resistance accompanied by reduced β-cell function (34). However, C-peptide remained stable among survivors with progressive glycemic deterioration at 1 year, suggesting that declining insulin sensitivity rather than loss of secretory capacity was the predominant mechanism in that cohort (41). These findings contrast with studies in which acute hyperglycemia was attributed mainly to peripheral insulin resistance rather than β-cell failure (26, 29).

Post-acute evidence indicated that metabolic recovery was not uniform. Insulin-resistance indices remained elevated at 3 and 6 months among adults without previous diabetes (25), while a proportion of children with MIS-C continued to exhibit pathological HOMA-IR or TyG values at 6 and 12 months despite normal HbA1c (28, 35). Persistent insulin resistance was also observed after viral clearance (29), approximately 2 months after hospitalization (31), among recovered Ghanaian adults (34), in long-COVID patients (42), and within the Bulgarian post-COVID metabolic disorder cohort (44–46). Gojda et al. found that acute insulin resistance resolved during follow-up, although hyperglycemia persisted in participants with insufficient insulin secretion (36). Glycemic abnormalities at 4 and 12 months were predicted by discharge glycemia, HOMA-IR, and inflammatory status (38), and approximately one-quarter of survivors with acute dysglycemia experienced glycemic progression at 1 year (41). Grubišić et al. distinguished transient stress hyperglycemia from persistent newly diagnosed diabetes through reassessment at 3 and 6 months (47). Conversely, the predominantly mild or asymptomatic infections evaluated by Goyal et al. were not associated with significant deterioration of insulin resistance, β-cell function, or glycemic category compared with non-infected participants (27).

Direct evidence of progression toward new-onset diabetes remained more limited than evidence of acute dysregulation or persistent insulin resistance. Goyal et al. directly evaluated glycemic progression but found no significant association with previous SARS-CoV-2 infection (27). Lui et al. documented progression from normoglycemia to prediabetes or diabetes, or from prediabetes to diabetes, in 23.8% of survivors reassessed at 1 year (41). The Tsvetkova study family characterized newly developed post-COVID disorders encompassing impaired fasting glucose, impaired glucose tolerance, insulin resistance, and diabetes phenotypes (44–46). Grubišić et al. identified a persistent newly diagnosed diabetes phenotype distinguished from transient stress hyperglycemia by higher insulin resistance during longitudinal follow-up (47). Other studies identified persistent insulin resistance, β-cell dysfunction, or sustained dysglycemia as potential diabetogenic signals but did not establish incident diabetes as a definitive longitudinal outcome (25, 28, 29, 31, 34, 36, 38).

Overall, the mapped evidence supports a temporal continuum in which acute inflammatory and metabolic stress promotes hyperglycemia through insulin resistance, with additional β-cell dysfunction in selected populations. Persistence of insulin resistance, glycemic abnormalities, or impaired insulin secretion after recovery may subsequently increase metabolic vulnerability; however, progression to overt diabetes was demonstrated in a smaller and more heterogeneous subset of studies. This pattern supports separate quantitative and narrative consideration of acute etiologic mechanisms, post-acute persistence, and prognostic progression.

### 3.4 Risk of bias in the included studies

Risk-of-bias assessments were reported separately according to study design and appraisal instrument; no composite score across tools was calculated. The design-specific judgments are presented in **Table 3**, panels A-F. The longitudinal etiological comparison evaluated using ROBINS-E was judged to raise some concerns overall (**Table 3A**). Goyal et al. incorporated pre-pandemic measurements, an uninfected comparison group, and adjustment for several baseline covariates. Nevertheless, the timing of SARS-CoV-2 infection was unavailable, exposure ascertainment based on serological or documented infection could have resulted in misclassification, inclusion required attendance at both study visits, and time-varying behavioral and metabolic confounding could not be fully addressed (27).

**Table 3:** Risk-of-bias assessments of the included studies according to study design and appraisal instrument.

Color key																												
Low risk / Yes	Some concerns / Moderate / Unclear									High or very high risk / No										Not applicable								
*Note.* Colors facilitate visualization but do not convert the instruments into a common scale. JBI checklist items are reported individually and are not summed into an overall numerical score.																												

A. ROBINS-E assessment																												
Study	D1		D2				D3				D4				D5				D6				D7				Overall	
Goyal et al. (27)	**SC**		**SC**				**SC**				**L**				**SC**				**L**				**SC**				**SC**	
*Note.* L, low risk; SC, some concerns; H, high risk; VH, very high risk. D1, bias due to confounding; D2, bias arising from measurement of the exposure; D3, bias in selection of participants into the study or analysis; D4, bias due to post-exposure interventions; D5, bias due to missing data; D6, bias arising from measurement of the outcome; D7, bias in selection of the reported result.																												

B. JBI cohort checklist																												
Study/family	C1	C2				C3		C4			C5			C6			C7		C8			C9		C10				C11
Chen et al. (25)	**NA**	**NA**				**Y**		**Y**			**N**			**N**			**Y**		**Y**			**Y**		**NA**				**Y**
Reiterer et al. (26)	**N**	**Y**				**Y**		**Y**			**N**			**N**			**N**		**NA**			**U**		**N**				**Y**
Calcaterra et al. (28, 35)	**NA**	**NA**				**Y**		**Y**			**N**			**N**			**Y**		**Y**			**Y**		**NA**				**N**
He et al. (29)	**N**	**Y**				**Y**		**Y**			**N**			**U**			**Y**		**Y**			**N**		**N**				**N**
Ilias et al. (30)	**N**	**Y**				**Y**		**Y**			**N**			**N**			**Y**		**NA**			**N**		**N**				**N**
Montefusco et al. (31)	**N**	**Y**				**Y**		**Y**			**N**			**N**			**Y**		**Y**			**N**		**N**				**U**
Gojda et al. (36)	**Y**	**Y**				**Y**		**Y**			**Y**			**N**			**Y**		**Y**			**N**		**N**				**Y**
Lui et al. (41), primary etiological analysis	**Y**	**Y**				**Y**		**Y**			**Y**			**U**			**Y**		**Y**			**U**		**N**				**Y**
*Note.* Y, yes; N, no; U, unclear; NA, not applicable. C1, groups similar and recruited from the same population; C2, exposure measured similarly; C3, exposure measured validly and reliably; C4, confounders identified; C5, strategies to address confounding; C6, outcome absent at baseline; C7, outcomes measured validly and reliably; C8, sufficient follow-up; C9, follow-up complete or losses described; C10, strategies for incomplete follow-up; C11, appropriate statistical analysis.																												

C. JBI analytical cross-sectional checklist																												
Study/family	X1		X2				X3				X4				X5				X6				X7				X8	
Soto et al. (33)	**Y**		**Y**				**Y**				**Y**				**N**				**N**				**Y**				**N**	
Adatsi et al. (34)	**Y**		**Y**				**Y**				**Y**				**Y**				**N**				**Y**				**N**	
Ahmed et al. (43)	**Y**		**Y**				**Y**				**Y**				**Y**				**N**				**Y**				**N**	
Tsvetkova et al. family (44–46)	**Y**		**Y**				**Y**				**Y**				**Y**				**U**				**Y**				**U**	
*Note.* Y, yes; N, no; U, unclear. X1, clear inclusion criteria; X2, participants and setting described; X3, exposure measured validly and reliably; X4, objective criteria for the condition; X5, confounders identified; X6, strategies to address confounding; X7, outcome measured validly and reliably; X8, appropriate statistical analysis.																												

D. JBI case-control checklist																												
Study	K1	K2				K3			K4			K5				K6			K7			K8		K9				K10
Al Masoodi et al. (42)	**N**	**N**				**Y**			**Y**			**Y**				**Y**			**Y**			**Y**		**U**				**U**
*Note.* Y, yes; N, no; U, unclear. K1, comparable groups; K2, appropriate matching; K3, same criteria for cases and controls; K4, valid and reliable exposure measurement; K5, exposure measured similarly; K6, confounders identified; K7, strategies to address confounding; K8, outcomes assessed validly and reliably; K9, meaningful exposure period; K10, appropriate statistical analysis.																												

E. QUIPS assessment																												
Study/result	Participation			Attrition				Prognostic-factor measurement					Outcome measurement					Confounding				Analysis/reporting				Overall		
Da Porto et al. (32)	**M**			**H**				**L**					**L**					**M**				**M**				**H**		
Liontos et al. (37)	**M**			**L**				**M**					**L**					**H**				**M**				**H**		
Andor et al. (38)	**H**			**H**				**L**					**L**					**H**				**H**				**H**		
Khan et al. (40)	**M**			**L**				**M**					**L**					**H**				**M**				**H**		
Grubišić et al. (47), acute prognosis	**M**			**L**				**M**					**L**					**M**				**M**				**M**		
Lui et al. (41), secondary 1-year progression analysis	**M**			**H**				**L**					**L**					**H**				**H**				**H**		
*Note.* L, low risk; M, moderate risk; H, high risk. The secondary analysis by Lui et al. (41) is an additional result-level assessment and is not counted as a separate study.																												

F. RoB 2 assessment																												
Study/result	D1				D2					D3					D4						D5				Overall			
Duijvelaar et al. (39), severe-hyperglycemia outcome	**L**				**L**					**L**					**L**						**SC**				**SC**			
Duijvelaar et al. (39), biomarker/HOMA outcomes	**L**				**L**					**SC**					**SC**						**SC**				**SC**			
*Note.* L, low risk; SC, some concerns; H, high risk. D1, bias arising from the randomization process; D2, bias due to deviations from intended interventions; D3, bias due to missing outcome data; D4, bias in measurement of the outcome; D5, bias in selection of the reported result. The two rows are outcome-specific judgments from the same randomized trial and do not represent separate studies.																												

Among the eight cohort studies or study families evaluated using the JBI checklist, metabolic outcomes were generally measured using objective laboratory methods; however, participant selection, inadequate control of confounding, and incomplete follow-up were recurrent limitations (**Table 3B**). Chen et al. retained all 64 enrolled participants through six months, but 73 of the original 149 patients declined follow-up, and the study lacked an uninfected comparison group (25). Reiterer et al. analyzed a large electronic health-record cohort, but HbA1c was unavailable for 2488 of 3854 participants, while the mechanistic classification of insulin resistance and beta-cell failure relied on selected biospecimen subgroups and an empirical C-peptide-glucose definition (26). The Calcaterra study family lacked an external comparison group. The extended report analyzed 38 participants at admission and at six and 12 months, but the relationship between the 30-patient initial report and the extended 38-patient cohort was not explicitly documented (28, 35). He et al. conducted several metabolic and omics analysis in small, nested subsets and used healthy controls with limited comparability to the hospitalized COVID-19 groups (29). Ilias et al. excluded patients transferred from the ward to intensive care and did not adequately adjust comparisons for differences related to age, disease severity, diabetes, or treatment (30). Montefusco et al. enrolled a substantial hospital cohort, but post-acute and mechanistic analysis were based on markedly smaller selected subgroups, including 57 participants with follow-up information (31). Gojda et al. included 26 of 37 enrolled participants in the complete-case analysis after excluding five participants with extreme outcome values and losing six during follow-up, although metabolic measurements were standardized and group comparisons were adjusted for age, sex, and body mass index (36). Lui et al. used non-COVID controls matched for age, sex, body mass index, and HbA1c for the primary comparison; nevertheless, only 43 of 52 COVID-19 survivors attended the one-year reassessment, and non-attenders had significantly higher baseline HbA1c (41).

The four analytical cross-sectional study families generally provided clear descriptions of their populations and used objective laboratory measurements, but none consistently controlled for the principal demographic, anthropometric, clinical, and treatment-related confounders (33, 34, 43–46). The corresponding domain-level judgments are presented in **Table 3C**. Soto et al. relied primarily on unadjusted comparisons between hospitalized patients and healthy controls (33). Adatsi et al. and Ahmed et al. identified relevant differences between comparison groups but did not estimate an adequately adjusted effect of previous or acute COVID-19 on concurrent glycemic dysregulation and insulin resistance (34, 43). In the overlapping Tsvetkova reports, adjustment was only partial, and the selection of post-COVID participants who had already developed metabolic abnormalities limited etiological interpretation (44–46). The 2026 Tsvetkova report also stated a total sample of 78 participants while describing two groups of 35 and 33 participants, respectively (44). The JBI case-control assessment is presented in **Table 3D**. The case-control study by Al Masoodi et al. did not explicitly match the comparison groups. Although its generalized linear models adjusted for sex, age, body mass index, smoking, maximum temperature, and oxygen saturation and the authors separately evaluated medication effects, substantial between-group differences in acute disease severity and treatment, the small sample, and the exclusively internal evaluation of the diagnostic model limited confidence in the findings (42).

Among the five primary prognostic studies evaluated using QUIPS, four were judged to have a high overall risk of bias and one a moderate risk (**Table 3E**). Da Porto et al. had complete stress-hyperglycemia and cytokine measurements for 150 of 200 consecutive participants, resulting in high concern regarding study attrition, together with moderate concern regarding residual confounding and model specification (32). Liontos et al. included a large retrospective cohort, but prognostic models were adjusted principally for age and sex; hyperglycemia analysis were additionally adjusted for corticosteroid exposure, leaving a high risk of residual confounding (37). Andor et al. selected 135 participants from an initial hospital cohort of 1980, with insufficient information concerning the derivation of the final longitudinal sample; the prediction models were not externally validated and did not comprehensively address relevant confounders (38). Khan et al. used a single baseline HOMA-IR measurement during critical illness and applied limited covariate adjustment; notably, HOMA-IR was not independently associated with mortality or organ dysfunction after adjustment for age and baseline disease severity (40). In contrast, the acute prognostic analysis by Grubišić et al. were judged to have a moderate overall risk because of prospective consecutive recruitment, predefined clinical outcomes, and parsimonious multivariable modeling. Nevertheless, deaths occurring within the first 24 hours and patients without adequate clinical documentation were excluded, and concerns remained regarding residual corticosteroid and pre-infection metabolic confounding, the use of HOMA-IR during acute illness, and the absence of external validation of data-derived ROC thresholds (47). The secondary prognostic analysis by Lui et al. was separately judged to have a high risk of bias because it included only 10 participants with glycemic progression, had differential loss to follow-up, and did not provide an adequately adjusted prognostic model (41).

The randomized imatinib comparison by Duijvelaar et al. was judged to raise some concerns rather than a high risk of bias (**Table 3F**). Randomization and double-blinding supported low risk for the randomization and intervention-deviation domains. However, the metabolic analysis were secondary and were not clearly prespecified; biomarker data were available for 318 of 385 randomized participants, ACE2 activity was evaluated in a matched subgroup of 76, and glucose, insulin, and C-peptide samples were typically obtained under nonfasting conditions. Consequently, concerns regarding missing data and outcome measurement were greater for the biomarker and HOMA-derived results than for the clinically defined severe-hyperglycemia outcome (39).

Overall, the principal threats to validity were residual confounding, selection of nested or nonrepresentative analytic samples, incomplete follow-up, reliance on surrogate measures of insulin resistance, and temporal ambiguity between acute illness, treatment exposure, hyperglycemia, and insulin resistance. These limitations should therefore be considered when interpreting causal associations, prognostic estimates, and any subsequent quantitative synthesis (**Table 3**, panels A-F).

### 3.5 Quantitative synthesis of prognostic clinical outcomes

Among the eligible prognostic outcomes, sufficient clinically and methodologically comparable data were available for quantitative synthesis only for short-term all-cause mortality and invasive mechanical ventilation. Exposure and comparator groups were defined using the study-specific criteria for acute hyperglycemia; the accompanying insulin-resistance or insulin-sensitivity assessments did not define the pooled participant groups. Exposure operationalization included new-onset hyperglycemia classified from glucose measurements obtained at admission and during hospitalization (31), an admission stress hyperglycemia ratio greater than 1.14 (32), incident grade 3 or higher hyperglycemia defined by the initiation of insulin therapy during hospitalization (39), and dysglycemia identified using repeated fasting glucose measurements and daily inpatient glycemic profiles (47). These definitions were considered clinically related manifestations of acute hyperglycemia but were not regarded as operationally interchangeable because they differed in threshold, timing, and consideration of previous glycemic status. Accordingly, the pooled RRs represent average prognostic associations across study-defined acute hyperglycemic phenotypes in studies that also assessed insulin resistance or insulin sensitivity; they do not estimate the effect of a participant-level composite phenotype, a universal glucose threshold, or a single uniform diagnostic entity. Exact exposure and outcome definitions are provided in **Supplementary Table 2**. Other clinical outcomes were retained in the systematic synthesis because fewer than three independent studies provided compatible data or because their outcome definitions and effect measures were not sufficiently comparable.

Compatible adjusted prognostic estimates were not sufficiently available for quantitative synthesis. Among the studies contributing to the meta- analysis, Da Porto et al. (32) was the only study that reported adjusted associations for the same dichotomized acute-hyperglycemia exposure and both meta-analyzed outcomes. Its multivariable models remained directionally consistent with the unadjusted findings for invasive mechanical ventilation and mortality after accounting for demographic, metabolic, inflammatory, and disease-severity-related variables. Montefusco et al. (31) reported age- and sex-adjusted estimates for broader respiratory-support outcomes, but not an adjusted mortality estimate corresponding to the pooled comparison, whereas Duijvelaar et al. (39) and Grubišić et al. (47) did not report compatible adjusted hyperglycemia–outcome estimates for the same contrasts. Therefore, an adjusted meta-analysis was not performed.

#### 3.5.1 Short-term all-cause mortality

Four independent studies contributed to the primary meta-analysis of short-term all-cause mortality (31, 32, 39, 47), comprising 1079 participants and 174 deaths (**Figure 2A**). Mortality occurred in 95 of 425 participants with an acute hyperglycemic phenotype and in 79 of 654 comparator participants. The acute hyperglycemic phenotype was associated with a higher risk of short-term all-cause mortality (RR 2.09, 95% CI 1.21 – 3.64; p = 0.024). Between-study heterogeneity was low to moderate (I^2^=31.3%; τ^2^=0.032; Q = 4.37, df=3, p=0.224). Although the 95% CI for the pooled average association excluded the null, the 95% prediction interval included it (0.96–4.56), indicating that the true association in a new comparable setting could range from little or no association to a substantially increased mortality risk.

**Figure 2:**
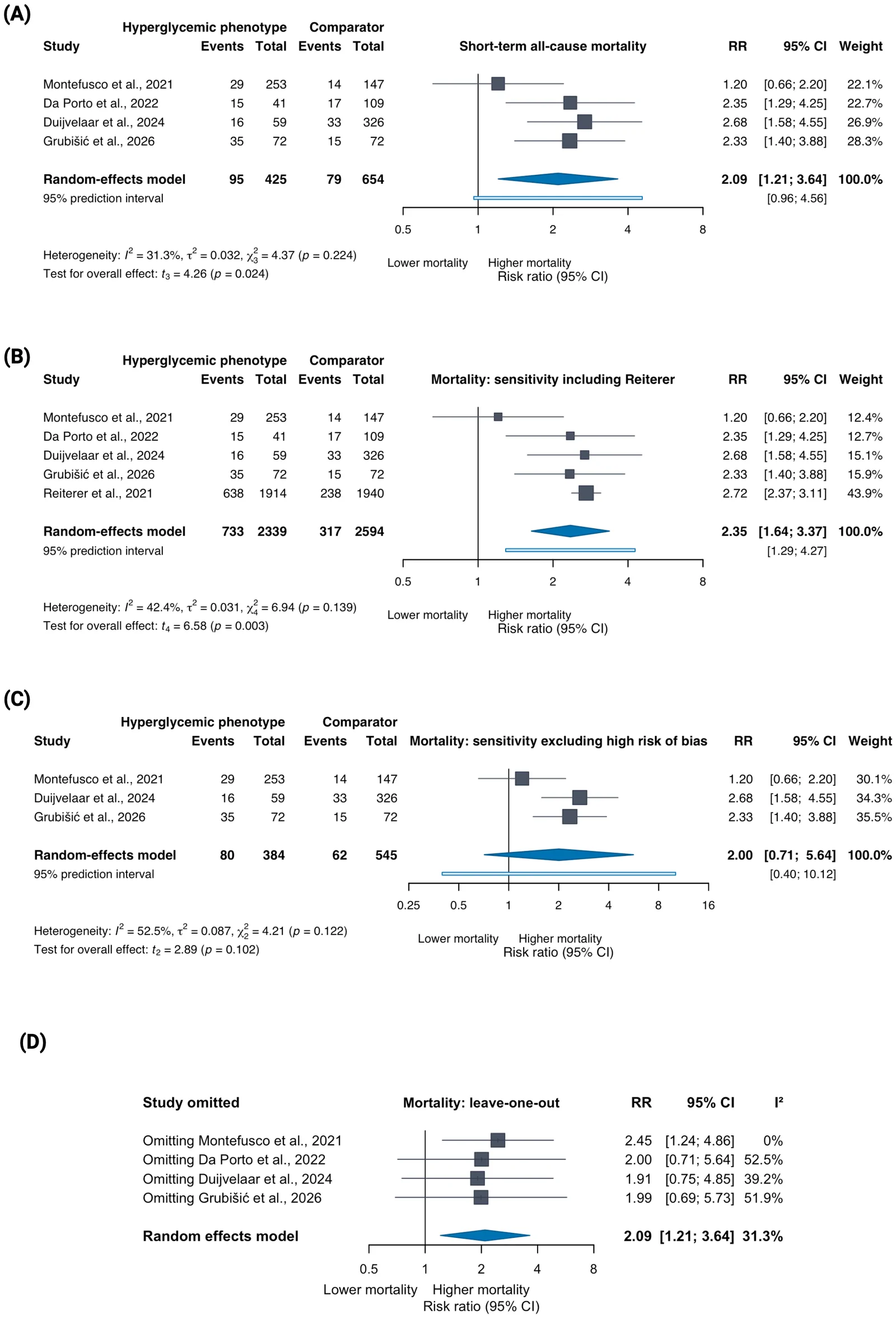
Primary and sensitivity meta-analysis of short-term all-cause mortality associated with an acute hyperglycemic phenotype. **(A)** Primary random-effects meta-analysis. **(B)** Sensitivity analysis including Reiterer et al., in which the hyperglycemic phenotype was classified using peak glucose >170 mg/dL recorded during hospitalization. **(C)** Sensitivity analysis excluding Da Porto et al., which was judged to be at high risk of bias. **(D)** Leave-one-out analysis showing the pooled estimate after sequential omission of each study. Risk ratios were pooled using inverse-variance random-effects models, with between-study variance estimated by restricted maximum likelihood and confidence intervals calculated using the modified Hartung-Knapp method. In **(A–C)**, squares represent individual study estimates, horizontal lines represent 95% confidence intervals, diamonds represent pooled estimates, and outlined bars represent 95% prediction intervals. In **(D)**, squares represent the pooled estimate obtained after each study omission, and the diamond represents the primary pooled estimate including all studies. Values greater than 1 indicate higher mortality among participants with an acute hyperglycemic phenotype. Event counts for Da Porto et al. and Grubišić et al. were reconstructed from the reported percentages and group denominators. CI, confidence interval; RR, risk ratio.

Adding Reiterer et al. (26), in which the hyperglycemic phenotype was defined using peak glucose recorded during hospitalization, increased the analysis to five studies and 4933 participants (**Figure 2B**). The direction and magnitude of the association remained consistent (RR 2.35, 95% CI 1.64 – 3.37; p = 0.003). Heterogeneity increased to I^2^ = 42.4% (τ^2^ = 0.031; Q = 6.94, df = 4, p = 0.139), while the prediction interval remained above the null value (1.29 – 4.27). After excluding Da Porto et al. (32), which was judged to be at high risk of bias, the pooled estimate remained similar in magnitude but became substantially less precise (k = 3; N = 929; RR 2.00, 95% CI 0.71 – 5.64; p = 0.102). Heterogeneity increased to I^2^ = 52.5% (τ^2^ = 0.087; Q = 4.21, df = 2, p = 0.122), and the prediction interval widened to 0.40 – 10.12 (**Figure 2C**).

In the leave-one-out analysis, the pooled estimates ranged from RR 1.91 to RR 2.45 and remained above the null value regardless of which study was omitted (**Figure 2D**). Exclusion of Montefusco et al. (31) produced an estimate of RR 2.45 (95% CI 1.24 – 4.86; I^2^ = 0%), whereas the confidence intervals included the null value after each of the other three omissions. No individual study reversed the direction or materially altered the magnitude of the association, although its precision was sensitive to the small number of contributing studies.

#### 3.5.2 Invasive mechanical ventilation

Three independent studies contributed to the primary meta-analysis of invasive mechanical ventilation (32, 39, 47), comprising 679 participants and 114 events (**Figure 3A**). Invasive ventilation occurred in 62 of 172 participants with an acute hyperglycemic phenotype and in 52 of 507 comparator participants. The acute hyperglycemic phenotype was associated with a higher risk of invasive mechanical ventilation (RR 3.64, 95% CI 1.74 – 7.61; p = 0.017). No between-study heterogeneity was observed (I^2^ = 0%; τ^2^ = 0; Q = 0.84, df = 2, p = 0.656). Because τ^2^ was estimated at zero, the 95% prediction interval was identical to the pooled confidence interval (1.74 – 7.61).

**Figure 3:**
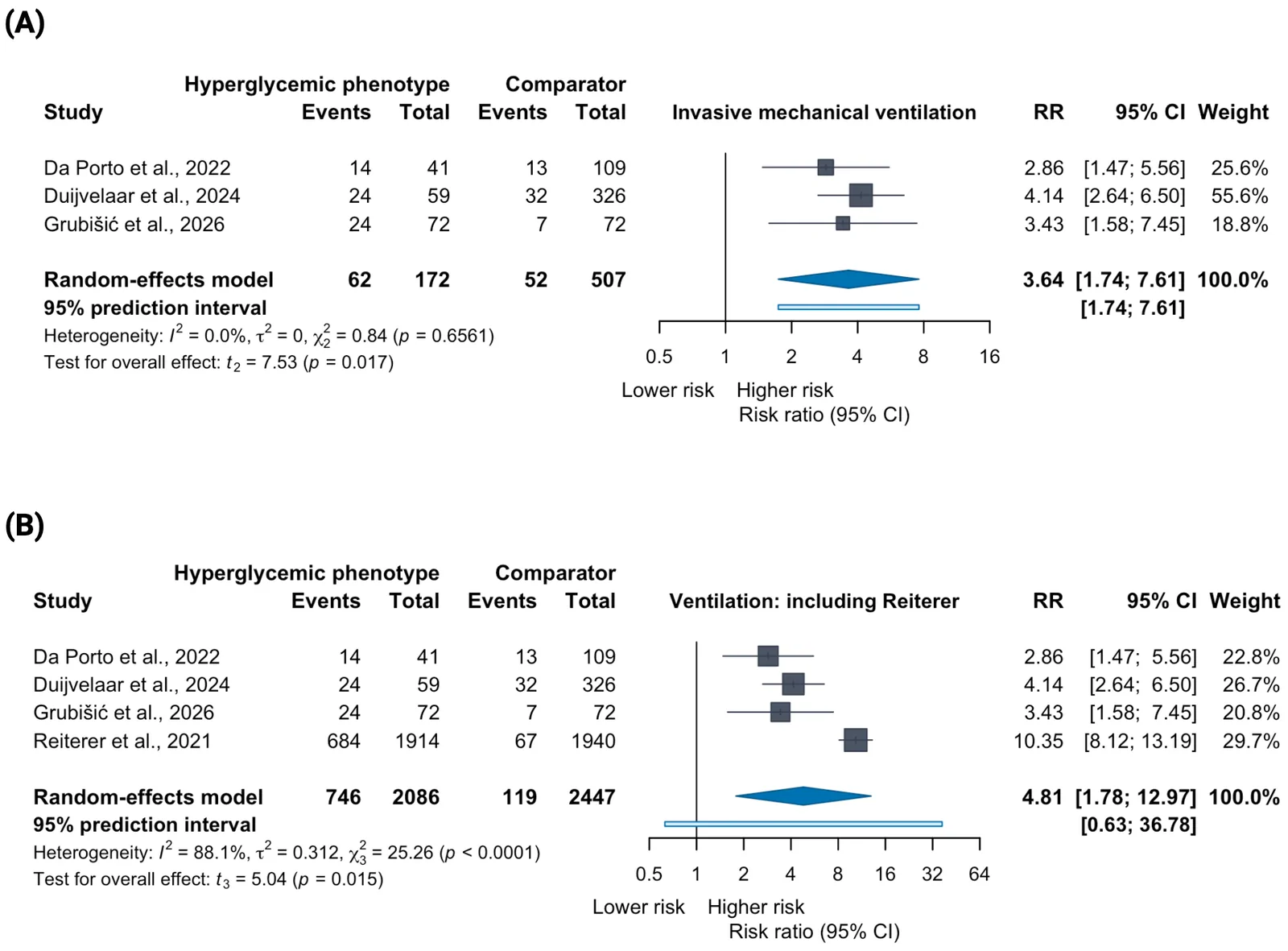
Primary and sensitivity meta-analysis of invasive mechanical ventilation associated with an acute hyperglycemic phenotype. **(A)** Primary random-effects meta-analysis. **(B)** Sensitivity analysis including Reiterer et al., in which the hyperglycemic phenotype was classified using peak glucose >170 mg/dL recorded during hospitalization. Risk ratios were pooled using inverse-variance random-effects models, with between-study variance estimated by restricted maximum likelihood and confidence intervals calculated using the modified Hartung-Knapp method. Squares represent individual study estimates, horizontal lines represent 95% confidence intervals, diamonds represent pooled estimates, and outlined bars represent 95% prediction intervals. Values greater than 1 indicate a higher risk of invasive mechanical ventilation among participants with an acute hyperglycemic phenotype. Event counts for Da Porto et al. and Grubišić et al. were reconstructed from the reported percentages and group denominators. CI, confidence interval; RR, risk ratio.

The sensitivity analysis incorporating Reiterer et al. (26) included four studies, 4,533 participants, and 865 events (**Figure 3B**). The pooled association remained compatible with a higher risk of invasive mechanical ventilation (RR 4.81, 95% CI 1.78–12.97; p = 0.015). However, heterogeneity increased substantially (I^2^ = 88.1%; τ^2^ = 0.312; Q = 25.26, df = 3, p < 0.0001), and the prediction interval crossed the null value (0.63–36.78). The study-specific RRs in the three primary studies were closely clustered (2.86, 4.14, and 3.43), whereas Reiterer et al. (26) reported a substantially larger and more precise association (RR 10.35, 95% CI 8.12–13.19). The exposed-group risk in Reiterer et al. (26) was similar to that observed in the primary studies (35.7% versus 33.3–40.7%), but the comparator-group risk was considerably lower (3.5% versus 9.7–11.9%), resulting in a larger relative contrast. Given its large sample size (n = 3,854) and narrow confidence interval, this discrepant estimate contributed strongly to Cochran’s Q and the increase in I^2^. Moreover, exposure was defined using peak glucose >170 mg/dL at any time during hospitalization rather than glycemic status established at or near baseline. Reiterer et al. was therefore retained as a sensitivity analysis rather than incorporated into the primary synthesis. A risk-of-bias exclusion analysis and an inferential leave-one-out analysis were not performed for invasive mechanical ventilation because removing any primary study would leave only two independent studies, below the prespecified minimum required for quantitative pooling.

### 3.6 Certainty of the evidence

The certainty of evidence was evaluated separately for the prognostic outcomes included in the meta-analysis and for the etiological outcomes synthesized without meta-analysis. The evidence for short-term all-cause mortality was rated as low certainty, whereas the evidence for invasive mechanical ventilation was rated as moderate certainty (**Table 4**). Both outcomes were downgraded one level for serious risk of bias because the meta- analysis were based on unadjusted estimates and the contributing studies presented residual confounding, participant-selection, missing-data, or exposure-measurement concerns (31, 32, 39, 47). Mortality was downgraded one additional level for serious inconsistency and uncertainty regarding transportability because its 95% prediction interval included the null association (0.96–4.56), despite directionally concordant study estimates and low-to-moderate statistical heterogeneity. No separate downgrade for imprecision was applied to mortality because the pooled confidence interval excluded the null and its lower limit remained clinically important; the prediction interval was considered within the inconsistency judgment to avoid double counting the same uncertainty. Invasive mechanical ventilation was not downgraded for inconsistency or imprecision because heterogeneity was absent and both its confidence and prediction intervals remained above the null association. Neither outcome was downgraded for indirectness. Publication bias could not be formally assessed because fewer than 10 studies contributed to each outcome.

**Table 4:** Summary of Findings and GRADE certainty for prognostic outcomes associated with study-defined acute hyperglycemic phenotypes.

Outcome	Studies and participants	Risk in comparator group	Risk with acute hyperglycemic phenotype	Effect estimate	Certainty and interpretation
Short-term all-cause mortality	4 studies; 1,079 participants	121 per 1,000	253 per 1,000 (146–440)	RR 2.09 (95% CI 1.21–3.64) 132 more per 1,000 (25–319 more)	⊕⊕◯◯ Low An acute hyperglycemic phenotype may be associated with higher short-term mortality. ^a–c, e, f^
Invasive mechanical ventilation	3 studies; 679 participants	103 per 1,000	373 per 1,000 (178–780)	RR 3.64 (95% CI 1.74–7.61) 270 more per 1,000 (76–678 more)	⊕⊕⊕◯ Moderate An acute hyperglycemic phenotype is probably associated with a greater risk of invasive ventilation. ^a, b, d–f^

Absolute risks in the exposed groups and absolute differences were calculated by applying the pooled unadjusted RRs to the aggregated event risks observed in the comparator groups: 79/654 for mortality and 52/507 for invasive ventilation. These values represent unadjusted prognostic associations and should not be interpreted as causal risk increases.

^a^ Observational prognostic evidence initially received a high-certainty rating in accordance with the GRADE approach for prognostic-factor questions.

^b^ Both outcomes were downgraded one level for serious risk of bias because the pooled effects were unadjusted and residual confounding, participant selection, missing data, and differences in exposure ascertainment remained possible. For mortality, this judgment integrated the corresponding JBI, QUIPS, and RoB 2 assessments; for invasive mechanical ventilation, it integrated the corresponding QUIPS and RoB 2 assessments. Tool-specific categories and item-level responses were retained and were not converted into a common numerical score.

^c^ Mortality was downgraded one additional level for serious inconsistency and uncertainty regarding transportability because the 95% prediction interval included the null association (0.96–4.56), despite directionally concordant study estimates and low-to-moderate heterogeneity (I^2^=31.3%).

^d^ Invasive mechanical ventilation was not downgraded for inconsistency because the study estimates were directionally concordant, heterogeneity was absent (I^2^=0%), and the 95% prediction interval remained above the null association (1.74–7.61).

^e^ Neither outcome was downgraded for indirectness or imprecision. The evidence directly addressed the prognostic question, the pooled confidence intervals excluded the null association, and their lower limits remained clinically important. For mortality, uncertainty represented by the prediction interval was incorporated into the inconsistency judgment and was not counted again under imprecision.

^f^ Publication bias could not be assessed statistically because fewer than 10 studies contributed to each outcome. The inability to perform formal testing was not, by itself, considered sufficient for downgrading.

CI, confidence interval; GRADE, Grading of Recommendations Assessment, Development and Evaluation; RR, risk ratio.

Evidence supporting an association between COVID-19 and insulin resistance or glycemic dysregulation was rated as low certainty because of serious risk of bias and inconsistency. Although most hospitalized or critically ill populations exhibited concurrent hyperglycemia and reduced insulin sensitivity (26, 29–33, 37, 39, 40, 47), findings were less consistent after predominantly mild or asymptomatic infection (27), and the relative contributions of insulin resistance and β-cell dysfunction varied across studies (25, 26, 29–31, 34, 36, 41).

Evidence concerning persistent post-COVID metabolic abnormalities was rated as very low certainty because of serious risk of bias, inconsistency, and imprecision (25, 27–29, 31, 34–36, 38, 41, 42, 44–47). Evidence concerning incident or progressive dysglycemia or diabetes was also rated as very low certainty because direct evidence was limited and findings differed among the four principal study families (27, 41, 44–47). Consequently, the evidence supports a possible continuum from acute metabolic disruption to persistent abnormalities but does not establish that SARS-CoV-2 infection independently causes insulin resistance or diabetes (**Table 5**).

**Table 5:** GRADE evidence profile for etiological outcomes synthesized without meta-analysis.

Etiological outcome	Evidence base	Summary of findings	GRADE judgments	Certainty and interpretation
COVID-19-associated insulin resistance or glycemic dysregulation	20 independent studies represented by 23 reports (25–47)	Most hospitalized or critically ill populations exhibited concurrent hyperglycemia and reduced insulin sensitivity. Findings were less consistent after mild or asymptomatic infection, and the relative contributions of peripheral insulin resistance and β-cell dysfunction varied.	Risk of bias: Serious^a^ Inconsistency: Serious^b^ Indirectness: Not serious Imprecision: Not serious Publication bias: Undetected^h^	⊕⊕◯◯ Low COVID-19 may be associated with insulin resistance or glycemic dysregulation, but confidence in the association is limited.
Persistent post-COVID metabolic abnormalities	12 primary study families and one supportive study family (25, 27–29, 31, 34–36, 38, 41–47)	Persistent abnormalities in insulin resistance, glycemia, or insulin secretion were documented from approximately 6 weeks to 12 months after COVID-19. However, some cohorts showed metabolic recovery, and controlled longitudinal comparisons were limited.	Risk of bias: Serious^a^ Inconsistency: Serious^c^ Indirectness: Not serious Imprecision: Serious^f^ Publication bias: Undetected^h^	⊕◯◯◯ Very low The evidence is very uncertain regarding the persistence of metabolic abnormalities after COVID-19.
Incident or progressive dysglycemia or diabetes	Four primary study families (27, 41, 44–47) and nine supportive study families (25, 28–31, 33–36, 38)	Glycemic progression or persistent newly diagnosed diabetes occurred in selected cohorts, whereas the only study with pre-pandemic measurements and a contemporaneous uninfected comparison group found no significant association with glycemic progression.	Risk of bias: Serious^a^ Inconsistency: Serious^d^ Indirectness: Serious^e^ Imprecision: Serious^g^ Publication bias: Undetected^h^	⊕◯◯◯ Very low The evidence is very uncertain regarding whether COVID-19 leads to incident or progressive dysglycemia or diabetes.

Etiological evidence was synthesized according to SWiM because differences in populations, comparators, metabolic measures, clinical phases, and follow-up periods precluded a valid pooled estimate. Participant totals were not aggregated because several publications used nested metabolic subcohorts or outcome-specific denominators. The paired reports by Calcaterra et al. (28, 35) and the partially overlapping reports by Tsvetkova et al. (44–46) were treated as single study families.

^a^ Downgraded one level for serious risk of bias based on the outcome-specific integration of ROBINS-E, QUIPS, JBI, and RoB 2 assessments. Participant selection, residual confounding, incomplete follow-up, absence of suitable non-infected comparison groups, and reliance on selected metabolic subcohorts were recurrent concerns across the contributing evidence. Instrument-specific categories and item-level responses were retained without calculating a cross-tool composite score.

^b^ Downgraded for serious inconsistency because results differed according to acute disease severity and because the reported metabolic mechanism varied between predominantly peripheral insulin resistance, combined insulin-resistance and secretory dysfunction, and absence of significant metabolic deterioration.

^c^ Downgraded for serious inconsistency because post-COVID trajectories ranged from persistent insulin resistance or glycemic abnormalities to partial or complete metabolic recovery.

^d^ Downgraded for serious inconsistency because controlled evidence did not demonstrate greater glycemic progression after predominantly mild or asymptomatic infection, whereas selected survivor cohorts reported progression or persistent newly diagnosed diabetes.

^e^ Downgraded for serious indirectness because several contributing studies evaluated selected populations with pre-existing or already developed metabolic abnormalities, or reported surrogate diabetogenic changes rather than prospectively confirmed incident diabetes.

^f^ Downgraded for serious imprecision because longitudinal analysis generally involved small or selected follow-up samples, and several studies did not report sufficiently precise comparative estimates.

^g^ Downgraded for serious imprecision because direct evidence was limited to four independent study families, progression events were few, and sufficiently precise controlled estimates were unavailable.

^h^ Formal assessment of publication bias was not possible because of the small number of directly comparable studies. No downgrade was applied in the absence of specific evidence of selective publication.

COVID-19, coronavirus disease 2019; GRADE, Grading of Recommendations Assessment, Development and Evaluation; SWiM, Synthesis Without Meta-analysis.

## 4 Discussion

### 4.1 Principal findings

This systematic review and meta-analysis integrated 20 independent studies represented by 23 reports to examine glycemic dysregulation and insulin resistance or insulin sensitivity across acute and post-acute COVID-19. The findings revealed different levels of certainty according to evidence function. COVID-19-associated insulin resistance or glycemic dysregulation was supported by low-certainty evidence, whereas persistent post-COVID metabolic abnormalities and progression toward dysglycemia or diabetes were supported by very-low-certainty evidence. In contrast, the mortality association was supported by low-certainty evidence, whereas the invasive mechanical ventilation association was supported by moderate-certainty evidence.

Acute hyperglycemia frequently coexisted with impaired insulin sensitivity, inflammatory activation, or altered insulin secretion among hospitalized and critically ill populations (26, 29–33, 47). However, the underlying mechanisms and subsequent metabolic trajectories were not uniform. Persistent abnormalities in insulin resistance or glycemic control were documented in several post-acute cohorts (25, 28, 29, 31, 34, 35, 38, 41, 42, 44–47), whereas other findings indicated metabolic recovery or no significantly greater deterioration after predominantly mild or asymptomatic infection (27, 36). Direct evidence of progression toward diabetes was more limited and discordant, ranging from no significant association in a controlled longitudinal comparison (27) to glycemic progression or persistent newly diagnosed diabetes in selected survivor populations (41, 44–47).

The quantitative synthesis showed that the acute hyperglycemic phenotype was associated with approximately twice the risk of short-term all-cause mortality (RR 2.09, 95% CI 1.21 – 3.64) across four studies (31, 32, 39, 47) and more than three times the risk of invasive mechanical ventilation (RR 3.64, 95% CI 1.74 – 7.61) across three studies (32, 39, 47). Sensitivity analysis including Reiterer et al. preserved the direction of both associations, although the substantial increase in heterogeneity for invasive ventilation highlighted the influence of exposure timing and the use of peak intrahospital glucose (26). Exclusion of the mortality study at high risk of bias and the leave-one-out analysis generally preserved the direction and magnitude of the association but substantially reduced its precision. Accordingly, the mortality meta-analysis supports an increased average prognostic association across the included studies, but its magnitude and reproducibility across clinical settings remain uncertain. Because the contributing studies used clinically related but operationally non-equivalent exposure definitions, the pooled RRs represent average class-level associations across study-defined acute hyperglycemic states. They should not be interpreted as the prognostic effect of a particular glucose threshold or as evidence that all included definitions identify an identical clinical entity.

Taken together, these findings support an integrated evidence framework linking glycemic dysregulation and insulin resistance rather than a uniform participant-level phenotype. Hyperglycemia formally defined the prognostic exposure and comparator groups, whereas insulin-resistance or insulin-sensitivity findings obtained within the same study populations or analytically linked subgroups provided the broader metabolic context. This framework extends previous syntheses that evaluated admission hyperglycemia, the stress hyperglycemia ratio, post-COVID diabetes, insulin resistance, or long COVID separately (2, 3, 8–10). Nevertheless, the findings remain associational: study-defined acute hyperglycemic phenotypes may identify patients at increased clinical risk, but the available evidence does not establish that insulin resistance contributed to the observed prognostic associations or that SARS-CoV-2 infection independently causes persistent insulin resistance or diabetes.

### 4.2 Integrated pathophysiological interpretation of acute metabolic dysregulation

Concurrent hyperglycemia and insulin resistance during acute COVID-19 are unlikely to result from a single metabolic lesion. A coherent interpretation is that systemic inflammatory stress, tissue-specific effects of SARS-CoV-2, host metabolic susceptibility, and treatment-related exposures converge on peripheral insulin action and β-cell compensatory capacity. Reiterer et al. characterized acute COVID-19-associated hyperglycemia as a predominantly insulin-resistant state accompanied by hyperinsulinemia, reduced adiponectin, and SARS-CoV-2 infectivity in adipose tissue (26). Da Porto et al. similarly linked stress hyperglycemia with greater insulin resistance and cytokine activation (32), whereas Soto et al. documented concomitant hyperglycemia, hyperinsulinemia, increased HOMA-IR, and oxidative and nitrosative stress (33). In the imatinib trial, attenuation of severe hyperglycemia was accompanied by changes consistent with reduced inflammatory signaling and improved peripheral glucose handling rather than increased insulin production (39). Together, these findings support inflammation-driven peripheral insulin resistance as an important component of acute metabolic dysregulation, although the clinical studies do not establish a single causal pathway.

Adipose tissue may represent an important interface between viral infection, inflammation, and impaired insulin action. Experimental and postmortem evidence has demonstrated SARS-CoV-2 RNA in adipocytes, productive infection of mature adipocytes, and inflammatory activation of adipose tissue-resident macrophages and bystander preadipocytes (5). At the molecular level, SARS-CoV-2 infection has also been associated with IRF1-dependent suppression of genes involved in the IRS–PI3K–AKT, mTOR, and MAPK components of insulin/IGF signaling in hepatic, adipose, pancreatic, and respiratory tissues (48). These observations provide biological plausibility for the reduced adiponectin, hyperinsulinemia, and insulin-resistance indices observed clinically (26, 32, 39). However, they do not demonstrate that adipose-tissue infection is necessary for acute metabolic dysregulation or that these molecular alterations persist after viral clearance.

The contribution of β-cell dysfunction appears more variable. Ilias et al. identified concurrent impairment of insulin sensitivity and insulin secretion during critical COVID-19 (30), whereas Montefusco et al. observed insulin resistance with β-cell hyperstimulation rather than uniform secretory failure (31). Gojda et al. subsequently showed that insulin sensitivity could improve during recovery while an insufficient insulin response remained relevant among patients with persistent glucose abnormalities (36). Experimental studies have demonstrated that SARS-CoV-2 can infect human pancreatic islets and β cells, reduce insulin content and glucose-stimulated insulin secretion, and induce β-cell injury or apoptosis (6, 49). Nevertheless, other carefully characterized pancreatic-tissue studies found ACE2 and TMPRSS2 predominantly in ducts and microvasculature rather than in β cells and detected viral proteins mainly in the ductal compartment (50, 51). Direct β-cell infection should therefore be regarded as a plausible contributory mechanism in some settings, but not as an established, necessary, or dominant explanation for COVID-19-associated hyperglycemia.

An integrated model is consequently more consistent with the evidence than a single-organ hypothesis. Inflammatory and counter-regulatory signaling can increase hepatic glucose production and reduce peripheral glucose uptake, while adipose dysfunction and impaired insulin signaling increase the demand placed on β cells. Oxidative stress and hyperglycemia may then amplify inflammatory and endothelial dysfunction, creating a self-reinforcing metabolic loop (32, 33). Glucocorticoid treatment can further reduce insulin sensitivity and challenge β-cell reserve, particularly in individuals with obesity, pre-existing dysglycemia, advanced age, or severe disease (1, 4). The resulting metabolic state may function simultaneously as a manifestation of metabolic stress and as a marker of limited metabolic reserve. However, the prognostic analysis evaluated study-defined acute hyperglycemia rather than this combined metabolic state; therefore, the integrated model provides biological context and should not be interpreted as evidence that a participant-level combination of hyperglycemia and insulin resistance caused the observed clinical outcomes. The interacting pathways and their potential transition toward post-acute metabolic abnormalities are summarized in **Figure 4**.

**Figure 4:**
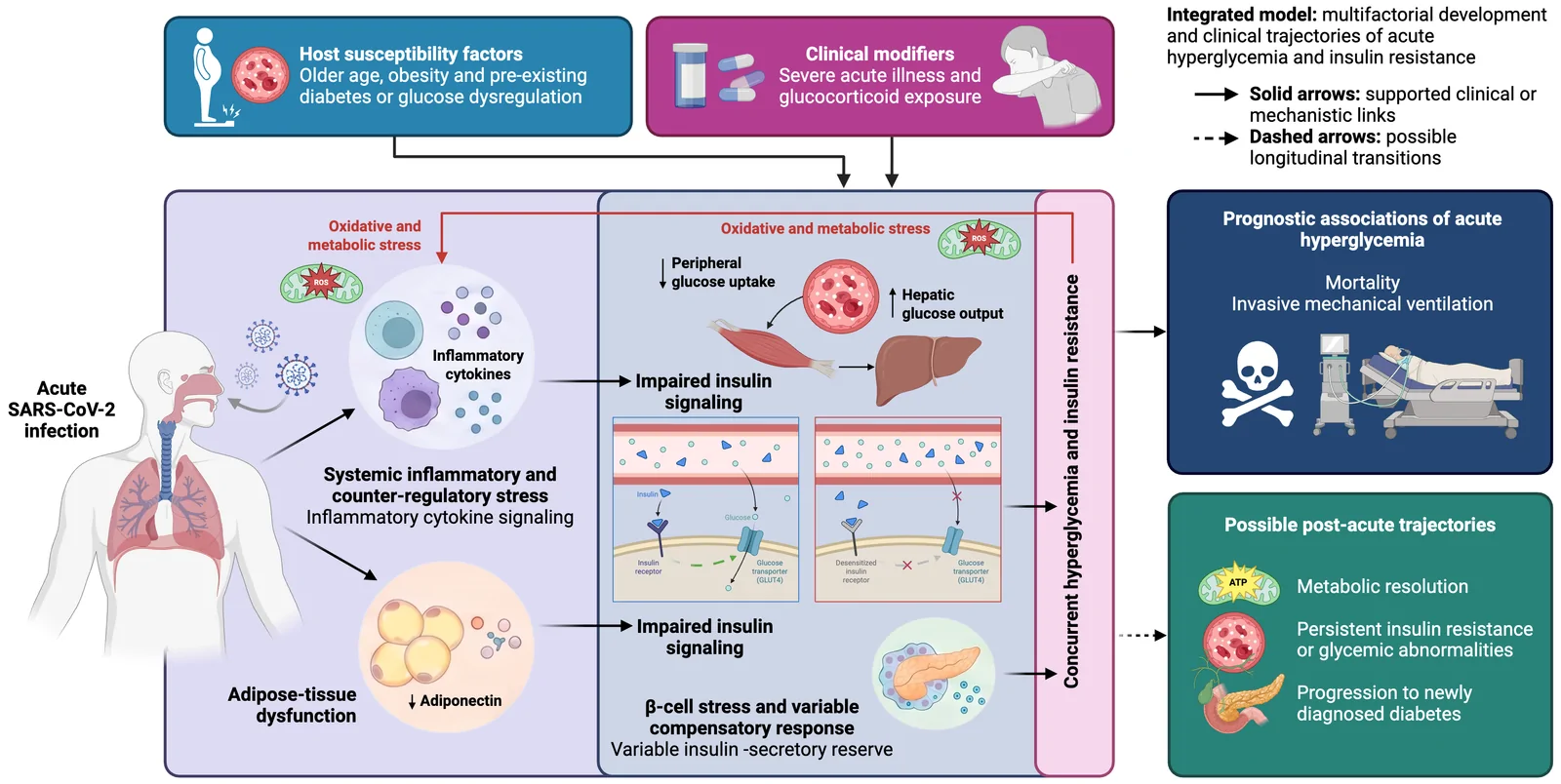
Integrated pathophysiological model linking acute hyperglycemia and insulin resistance in COVID-19. Host susceptibility factors and clinical modifiers may interact with SARS-CoV-2-associated systemic inflammation, counter-regulatory stress, adipose-tissue dysfunction, impaired insulin signaling, and variable β-cell compensation to produce concurrent hyperglycemia and insulin resistance in some patients. In the reviewed prognostic studies, study-defined acute hyperglycemic phenotypes were associated with mortality and invasive mechanical ventilation; insulin-resistance or insulin-sensitivity measurements provided metabolic context rather than defining a standardized participant-level combined exposure. Possible post-acute trajectories range from metabolic resolution to persistent insulin resistance or glycemic abnormalities and progression to newly diagnosed diabetes. The feedback arrow represents the potential amplification of systemic inflammation by oxidative and metabolic stress. Solid arrows indicate supported clinical or mechanistic relationships, whereas dashed arrows represent possible longitudinal transitions for which causal certainty remains limited. Created in BioRender. Carrillo, J (2026). https://BioRender.com/9filpbt.

### 4.3 Prognostic significance of acute hyperglycemic phenotypes

Study-defined acute hyperglycemic phenotypes were associated with both major prognostic outcomes evaluated in this review. In the primary analysis, participants meeting the study-specific hyperglycemia definitions had approximately twice the risk of short-term all-cause mortality and more than three times the risk of invasive mechanical ventilation compared with the corresponding normoglycemic or non-severely hyperglycemic groups (31, 32, 39, 47). The larger association observed for ventilation may reflect the close relationship between acute metabolic stress and respiratory disease severity, although differences between outcome definitions and contributing study populations preclude direct comparison of the two pooled estimates. These findings are consistent with previous meta- analysis of admission hyperglycemia and the stress hyperglycemia ratio in COVID-19 (2, 3) but extend that evidence by restricting the quantitative synthesis to studies that also assessed insulin resistance or insulin sensitivity.

The mortality association remained directionally stable across sensitivity analysis, but its precision depended on the small number of contributing studies. Exclusion of the study judged to be at high risk of bias produced a similar point estimate, whereas the confidence interval widened and crossed the null because only three studies remained (31, 39, 47). Likewise, the leave-one-out estimates consistently indicated increased risk, with no individual study reversing the direction of association, although statistical significance was not retained after several omissions. The pooled 95% CI of 1.21–3.64 quantifies uncertainty around the average association across the included studies and excludes the null value. In contrast, the 95% prediction interval of 0.96–4.56 incorporates between-study variability and estimates the range of true associations that might be observed in a new comparable setting. Its inclusion of 1.0 therefore indicates that a future setting could show little or no association, even though the average pooled association was statistically significant. These findings are not contradictory: they support an increased average mortality association while indicating that its magnitude may not be uniform across populations or clinical settings. Because only four studies contributed to the analysis, the prediction interval itself should also be interpreted cautiously. This uncertainty contributed to the low-certainty GRADE rating for mortality. For invasive mechanical ventilation, the three primary studies produced concordant estimates, no detected heterogeneity, and a prediction interval entirely above the null association (32, 39, 47); nevertheless, uncertainty regarding heterogeneity remains because only three studies were available.

Including Reiterer et al. strengthened the pooled mortality association but markedly increased heterogeneity in the analysis of invasive mechanical ventilation (26). This increase was attributable to both statistical and clinical differences. The ventilation RRs from the three primary studies were closely clustered between 2.86 and 4.14, whereas Reiterer et al. (26) reported a substantially larger and more precise estimate (RR 10.35). Although the ventilation risk among participants classified as hyperglycemic was similar across studies, the comparator-group risk in Reiterer et al. (26) was considerably lower, producing a larger relative contrast. Because Reiterer et al. (26) also contributed a substantially larger sample and a narrow confidence interval, its discrepant estimate had a strong influence on the heterogeneity statistic. In addition, exposure classification was based on peak glucose values recorded at any time during hospitalization rather than glycemic status established at or near baseline. Peak in-hospital hyperglycemia may reflect not only underlying metabolic vulnerability but also evolving disease severity, glucocorticoid exposure, nutritional support, or other consequences of intensive care, introducing temporal ambiguity and possible reverse temporality. These differences support retaining Reiterer et al. (26) as a sensitivity analysis rather than combining it with the primary, temporally anchored exposure definitions.

The pooled RRs should therefore be interpreted as prognostic associations rather than causal effects. Acute hyperglycemia has long been recognized as a marker of adverse outcomes among hospitalized patients, particularly when newly detected rather than occurring in established diabetes (52). Measures such as the stress hyperglycemia ratio may improve prognostic interpretation by accounting for background glycemia and distinguishing relative acute deterioration from chronically elevated glucose (53). However, the exposure definitions used by the included studies were not uniform, and the pooled estimates were derived from unadjusted event data. Residual confounding by age, obesity, pre-existing diabetes, acute disease severity, glucocorticoid treatment, and clinical management remains likely. Accordingly, study-defined acute hyperglycemic phenotypes may serve as risk-stratification signals, but the present evidence does not establish that routine measurement of insulin-resistance indices adds prognostic value or that glucose-lowering treatment guided by concurrent metabolic measurements improves COVID-19 outcomes.

### 4.4 Post-acute metabolic trajectories and newly diagnosed diabetes

The post-acute evidence did not support a single metabolic trajectory after COVID-19. Instead, the included studies described three possible courses: resolution of the acute disturbance, persistence of insulin resistance or glycemic abnormalities, and progression toward newly diagnosed diabetes. These trajectories could not be combined quantitatively because the studies differed in baseline metabolic characterization, comparator selection, follow-up duration, and the indices used to assess insulin resistance, insulin sensitivity, and glucose regulation. Importantly, changes in HOMA-derived measures, the triglyceride–glucose index, dynamic insulin-sensitivity indices, or diagnostic glycemic categories should not be interpreted as interchangeable manifestations of the same outcome.

Evidence of metabolic resolution or absence of measurable progression was observed in several settings. In participants with predominantly mild or asymptomatic infection and available pre-pandemic measurements, SARS-CoV-2 infection was not significantly associated with glycemic progression or worsening HOMA-IR categories compared with non-infected participants (27). Following severe COVID-19, insulin sensitivity improved during follow-up in another cohort, suggesting that at least part of the acute insulin-resistant state may be reversible (36). Marker-specific discordance was also apparent: over six months, the triglyceride–glucose index increased in adults without previous diabetes, whereas HOMA-IR did not change significantly (25). These findings indicate that apparent persistence depends partly on the metabolic construct and measurement method selected.

Conversely, persistent or progressive abnormalities were identified in clinically selected subgroups. Montefusco et al. documented sustained abnormalities in glucose regulation and continuous glucose-monitoring profiles after acute COVID-19, with persistent hyperglycemic alterations and progression to overt diabetes occurring in a subset of participants (31). Persistent abnormalities in HOMA-IR and the triglyceride–glucose index were also reported during follow-up after multisystem inflammatory syndrome (35). At one year, Lui et al. observed glycemic-category progression in 10 of 42 participants and deterioration in the Matsuda insulin-sensitivity index, although HOMA-IR remained statistically unchanged and the original control group was not reassessed (41). Individuals with long COVID showed greater HOMA2-IR and lower HOMA2-%S than recovered individuals without long COVID, but differences in acute severity and treatment exposure limited attribution specifically to persistent SARS-CoV-2 effects (42). Grubišić et al. further demonstrated the importance of distinguishing transient stress hyperglycemia from newly diagnosed diabetes during follow-up rather than treating all acute hyperglycemia as a permanent metabolic diagnosis (47).

These heterogeneous trajectories are consistent with broader epidemiological evidence showing an increased incidence of diabetes after COVID-19 (8). In a large cohort of United States veterans, survivors of COVID-19 had higher post-acute risks and burdens of incident diabetes and antihyperglycemic medication use than contemporary and historical controls, with risk increasing according to acute disease severity (54). However, increased post-infection diagnoses do not necessarily represent irreversible diabetes directly caused by SARS-CoV-2. In a hospital cohort, approximately 41% of survivors with diabetes first diagnosed during COVID-19 admission subsequently regressed to normoglycemia or prediabetes, supporting acute stress-related insulin resistance or the unmasking of previously unrecognized metabolic susceptibility in a substantial proportion of patients (55).

Taken together, the evidence suggests that COVID-19 may precipitate acute insulin resistance and expose limited metabolic reserve, while persistent insulin resistance, glycemic abnormalities, or newly diagnosed diabetes develop in only a subset of patients. The relative contributions of direct viral effects, inflammatory stress, glucocorticoid exposure, pre-existing but undiagnosed diabetes or glucose abnormalities, adiposity, behavioral changes, and intensified post-COVID medical surveillance remain difficult to separate. Therefore, the available evidence supports post-acute metabolic surveillance in clinically vulnerable patients but does not establish that every new diabetes diagnosis following COVID-19 is causally attributable to SARS-CoV-2.

### 4.5 Clinical and research implications

Acute hyperglycemia should be interpreted clinically as a risk marker rather than as evidence that all affected participants have concurrent insulin resistance. Its associations with mortality and invasive mechanical ventilation support careful assessment of glucose abnormalities in hospitalized patients with COVID-19, particularly among those with obesity, previous diabetes or glucose abnormalities, severe disease, or glucocorticoid exposure (31, 32, 39, 47). Admission glucose should be interpreted alongside previous glycemic status and the timing of treatment. Current hospital-care recommendations support measuring HbA1c in patients with diabetes or newly recognized hyperglycemia when no result from the preceding three months is available, because this helps distinguish chronic glucose elevation from acute stress- or treatment-related hyperglycemia and informs discharge planning (56, 57). Nevertheless, the present review does not establish that routine measurement of HOMA-IR, HOMA2-IR, TyG, or other insulin-resistance indices improves clinical decisions, nor that more intensive glucose lowering guided by concurrent insulin-resistance measurements improves COVID-19 outcomes.

Post-discharge assessment should similarly reflect the heterogeneity of the observed trajectories. Acute glucose abnormalities may resolve, persist, or progress, and a diabetes diagnosis made during severe infection may represent previously unrecognized disease, transient stress hyperglycemia, glucocorticoid-associated hyperglycemia, or a genuinely persistent post-COVID metabolic disorder (25, 27, 31, 36, 41, 42, 47). Patients with newly detected hyperglycemia, substantial insulin requirements, severe acute disease, obesity, or other metabolic risk factors may therefore benefit from reassessment after recovery using established diagnostic tests. However, inpatient hyperglycemia alone should not be assumed to represent permanent diabetes without confirmation after the acute inflammatory and treatment-related disturbances have subsided.

Future etiological studies require pre-infection or early baseline measurements, contemporaneous non-infected comparators, serial follow-up, and standardized assessment times. They should record HbA1c, adiposity, previous diabetes risk, glucocorticoid dose and duration, nutritional support, glucose-lowering therapy, and acute disease severity. Insulin-resistance and insulin-sensitivity measures should be selected according to the biological construct of interest and should not be treated as interchangeable. Studies evaluating progression to diabetes should distinguish transient stress hyperglycemia, newly recognized pre-existing diabetes, and persistent incident disease through repeated diagnostic testing. Reassessment of comparator groups is also essential to separate infection-associated changes from background metabolic progression and pandemic-related behavioral effects.

Future prognostic studies should define exposure before the occurrence of clinical outcomes and evaluate whether combining acute glucose measurements with HbA1c, relative hyperglycemia, and validated insulin-resistance markers provides incremental predictive value beyond established clinical severity indicators. Models should report calibration, discrimination, clinical utility, and external validation rather than statistical association alone. Individual-participant data would be particularly valuable for constructing a reproducible classification that explicitly combines hyperglycemia and insulin resistance and for examining effect modification by pre-existing diabetes, obesity, age, sex, glucocorticoid exposure, and acute disease severity. Ultimately, prospective intervention studies are required to determine whether identifying or treating such combined metabolic abnormalities changes acute clinical or long-term glycemic outcomes.

### 4.6 Strengths and limitations

A major strength of this review is the integration of etiological, longitudinal, mechanistic, and prognostic evidence within a single acute-to-post-acute metabolic framework. Rather than pooling biologically distinct outcomes, quantitative synthesis was restricted to clinically comparable mortality and invasive-ventilation data, while non-combinable metabolic evidence was synthesized systematically according to SWiM principles. Reports derived from the same or overlapping populations were organized as study families, preventing duplicate participant counting while preserving complementary outcomes and follow-up periods, particularly across the three related reports from Tsvetkova et al. (44–46). The analysis also incorporated random-effects models with REML estimation, Hartung–Knapp adjustment, prediction intervals, prespecified sensitivity analysis, and leave-one-out evaluation.

A further strength was the source-level audit of extracted data. Published, reconstructed, and non-combinable results were explicitly differentiated, and discrepancies were documented rather than corrected without traceability. This process identified an internally inconsistent HOMA-IR result (34), incompatible probability values and confidence intervals (32), differences between total-cohort and biomarker-substudy denominators (39), and event counts reconstructed from published percentages (47). Risk of bias was evaluated using design-appropriate tools, and certainty was assessed separately for prognostic and etiological outcomes. These procedures reduced the risk that heterogeneous or internally inconsistent metabolic measurements would be converted into apparently precise pooled estimates.

Several limitations nevertheless affect interpretation. The primary mortality and ventilation meta- analysis included only four and three independent studies, respectively (31, 32, 39, 47). Consequently, pooled estimates and heterogeneity measures remained imprecise, prediction intervals were wide, and formal assessment of small-study effects, meta-regression, and reliable subgroup analysis was not possible. The pooled RRs were reconstructed from unadjusted event data and therefore represent total prognostic associations rather than effects independent of confounding. Compatible adjusted estimates for the same exposure–outcome contrasts were available from only one contributing study, precluding an adjusted meta-analysis; estimates based on different outcomes, effect measures, covariate sets, or model specifications were not combined. Although the available adjusted associations were directionally consistent with the pooled findings, residual confounding by age, obesity, pre-existing diabetes, disease severity, glucocorticoid exposure, and differences in clinical management remains likely. Exposure definitions were clinically related but not operationally uniform, encompassing new-onset hyperglycemia assessed from serial glucose measurements, an admission stress hyperglycemia ratio, incident severe hyperglycemia requiring insulin therapy, and dysglycemia identified through repeated inpatient glucose profiles. This supported a cautious synthesis at the level of a broader acute hyperglycemic exposure class, but the resulting RRs cannot define the prognostic effect of a universal glucose threshold or a single diagnostic category. The small number of studies precluded subgroup analysis or meta-regression according to exposure definition, measurement timing, diabetes status, or corticosteroid use. Some exposures were determined at admission, whereas severe hyperglycemia requiring insulin therapy was identified during hospitalization, creating greater temporal ambiguity. Reiterer et al., which classified exposure using peak glucose recorded at any time during hospitalization, was restricted to sensitivity analysis because this definition was least temporally aligned with the primary exposure framework and was particularly susceptible to evolving disease severity, treatment-related hyperglycemia, and reverse temporality (26).

The etiological evidence was limited predominantly by observational designs, small or clinically selected samples, inconsistent baseline characterization, and the absence of contemporaneously reassessed controls in several longitudinal studies (25, 31, 35, 41, 42). HOMA-IR, HOMA2-IR, HOMA2-%S, Matsuda, TyG, continuous glucose-monitoring outcomes, and diagnostic glycemic categories represented related but non-equivalent constructs and could not be combined validly. Newly diagnosed diabetes after COVID-19 could not always be distinguished from previously unrecognized diabetes, transient stress hyperglycemia, or glucocorticoid-associated hyperglycemia. Most evidence was also derived from early-pandemic and hospitalized populations, limiting generalizability across later viral variants, vaccination status, contemporary treatments, mild community-managed infection, and more diverse populations.

Finally, glycemic dysregulation and insulin resistance were integrated at the evidence level, but the included studies did not consistently classify participants using a standardized combination of both components. Clinical events were generally reported according to glycemic categories rather than participant-level combined metabolic status. The present meta- analysis therefore quantify the prognostic association of study-defined acute hyperglycemic phenotypes in studies that also evaluated insulin resistance or insulin sensitivity; they do not estimate the effect of a uniform composite exposure. This distinction limits causal and diagnostic interpretation but preserves fidelity to the available evidence.

## 5 Conclusion

This systematic review integrates evidence on glycemic dysregulation and insulin resistance as related features of metabolic disruption during COVID-19. Quantitative synthesis showed that study-defined acute hyperglycemic phenotypes, in studies that also evaluated insulin resistance or insulin sensitivity, were associated with approximately twice the risk of short-term all-cause mortality and more than three times the risk of invasive mechanical ventilation. Certainty was low for mortality and moderate for invasive mechanical ventilation; moreover, the mortality prediction interval included the null association, indicating that the pooled average may not apply uniformly across comparable clinical settings. These estimates are prognostic and associative and should not be interpreted as evidence that insulin resistance contributed to the risk associated with the study-defined hyperglycemic exposure or that either abnormality directly caused the outcomes. The etiologic evidence suggests that SARS-CoV-2 infection may precipitate acute insulin resistance and glucose dysregulation, but subsequent trajectories are heterogeneous. Metabolic abnormalities resolve in some patients, persist in others, and progress toward newly diagnosed diabetes in a smaller subset. Current evidence cannot fully separate direct infection-related effects from inflammatory stress, glucocorticoid exposure, acute disease severity, adiposity, or previously unrecognized metabolic disease.

Accordingly, acute hyperglycemia should prompt assessment of previous glycemic status and appropriate post-recovery reassessment, particularly in metabolically vulnerable patients. Future prospective studies should establish pre-infection metabolic status, use contemporaneous controls, apply standardized participant-level definitions when explicitly combining hyperglycemia and insulin resistance, and determine whether assessment or treatment strategies guided by these metabolic abnormalities improve acute clinical outcomes or prevent persistent metabolic disease.

## Data Availability

The original contributions presented in the study are included in the article/**Supplementary Material**. Further inquiries can be directed to the corresponding authors.
