# Supplementary material for: Glycemic dysregulation and insulin resistance across acute and post-acute COVID-19: a systematic review and meta-analysis of etiologic and prognostic evidence
Source: Front Endocrinol (Lausanne). 2026 Oct 14;17:1975728. doi: 10.3389/fendo.2026.1975728 (PMC13713293; doi:10.3389/fendo.2026.1975728)
Supplement: Supplementary file 1 [file Table1.docx]

Supplementary Material

**Supplementary Table 1.** Complete electronic search strategies and records retrieved

| **Database** | **Search set** | **Search strategy** | **Records retrieved** |
| --- | --- | --- | --- |
| **PubMed** |  |  | **94** |
|  | **S1** | “Insulin resistance” OR “Insulin Sensitivity” OR “Resistance, Insulin” OR “Sensitivity, Insulin” |  |
|  | **S2** | “Hyperglycemia” OR “Hyperglycemia, Postprandial” OR “Hyperglycemias” OR “Hyperglycemias, Postprandial” OR “Postprandial Hyperglycemia” OR “Postprandial Hyperglycemias” |  |
|  | **S3** | “2019 nCoV Disease” OR “2019 nCoV Infection” OR “2019 Novel Coronavirus Disease” OR “2019 Novel Coronavirus Epidemic” OR “2019 Novel Coronavirus Infection” OR “2019 Novel Coronavirus Outbreak” OR “2019 Novel Coronavirus Pandemic” OR “2019 Novel Coronavirus Pneumonia” OR “2019-20 China Pneumonia Outbreak” OR “2019-20 Wuhan Coronavirus Outbreak” OR “2019-nCoV Acute Respiratory Disease” OR “2019-nCoV Disease” OR “2019-nCoV Diseases” OR “2019-nCoV Epidemic” OR “2019-nCoV Infection” OR “2019-nCoV Infections” OR “2019-nCoV Outbreak” OR “2019-nCoV Pandemic” OR “2019-nCoV Pneumonia” OR “2019-New Coronavirus Epidemic” OR “2019-Novel Coronavirus (2019-nCoV) Infection” OR “2019-Novel Coronavirus Pneumonia” OR “Coronavirus Disease 19” OR “Coronavirus Disease 2019” OR “Coronavirus Disease-19” OR “COVID 19” OR “COVID 19 Pandemic” OR “COVID 19 Virus Disease” OR “COVID 19 Virus Infection” OR “COVID-19” OR “COVID-19 Pandemic” OR “COVID-19 Pandemics” OR “COVID-19 Virus Disease” OR “COVID-19 Virus Diseases” OR “COVID-19 Virus Infection” OR “COVID-19 Virus Infections” OR “COVID19” OR “Disease 2019, Coronavirus” OR “Disease, 2019-nCoV” OR “Disease, COVID-19 Virus” OR “Infection, 2019-nCoV” OR “Infection, COVID-19 Virus” OR “Infection, SARS-CoV-2” OR “Novel Coronavirus Pneumonia” OR “Pandemic, COVID-19” OR “SARS Coronavirus 2 Infection” OR “SARS CoV 2 Infection” OR “SARS-CoV-2 Infection” OR “SARS-CoV-2 Infections” OR “Severe Acute Respiratory Syndrome Coronavirus 2 Infection” OR “Virus Disease, COVID-19” OR “Virus Infection, COVID-19” OR “Wuhan Coronavirus Epidemic” OR “Wuhan Coronavirus Infection” OR “Wuhan Coronavirus Outbreak” OR “Wuhan Coronavirus Pandemic” OR “Wuhan Coronavirus Pneumonia” OR “Wuhan Seafood Market Pneumonia” OR “SARS-CoV-2” OR “Wuhan Coronavirus” OR “Coronavirus, Wuhan” OR “Wuhan Seafood Market Pneumonia Virus” OR “Coronavirus Disease 2019 Virus” OR “Severe Acute Respiratory Syndrome Coronavirus 2” OR “SARS Coronavirus 2” OR “Coronavirus 2, SARS” OR “COVID19 Virus” OR “COVID19 Viruses” OR “Virus, COVID19” OR “Viruses, COVID19” OR “2019-nCoV” OR “SARS-CoV-2 Virus” OR “SARS CoV 2 Virus” OR “SARS-CoV-2 Viruses” OR “Virus, SARS-CoV-2” OR “COVID-19 Virus” OR “COVID 19 Virus” OR “COVID-19 Viruses” OR “Virus, COVID-19” OR “2019 Novel Coronavirus” OR “2019 Novel Coronaviruses” OR “Coronavirus, 2019 Novel” OR “Novel Coronavirus, 2019” |  |
|  | **FS** | **(S1) AND (S2) AND (S3)** |  |
| **Scopus** |  |  | **293** |
|  | **S1** | “Insulin resistance” OR “Insulin Sensitivity” OR “Resistance, Insulin” OR “Sensitivity, Insulin” |  |
|  | **S2** | “Hyperglycemia” OR “Hyperglycemia, Postprandial” OR “Hyperglycemias” OR “Hyperglycemias, Postprandial” OR “Postprandial Hyperglycemia” OR “Postprandial Hyperglycemias” |  |
|  | **S3** | “2019 nCoV Disease” OR “2019 nCoV Infection” OR “2019 Novel Coronavirus Disease” OR “2019 Novel Coronavirus Epidemic” OR “2019 Novel Coronavirus Infection” OR “2019 Novel Coronavirus Outbreak” OR “2019 Novel Coronavirus Pandemic” OR “2019 Novel Coronavirus Pneumonia” OR “2019-20 China Pneumonia Outbreak” OR “2019-20 Wuhan Coronavirus Outbreak” OR “2019-nCoV Acute Respiratory Disease” OR “2019-nCoV Disease” OR “2019-nCoV Diseases” OR “2019-nCoV Epidemic” OR “2019-nCoV Infection” OR “2019-nCoV Infections” OR “2019-nCoV Outbreak” OR “2019-nCoV Pandemic” OR “2019-nCoV Pneumonia” OR “2019-New Coronavirus Epidemic” OR “2019-Novel Coronavirus (2019-nCoV) Infection” OR “2019-Novel Coronavirus Pneumonia” OR “Coronavirus Disease 19” OR “Coronavirus Disease 2019” OR “Coronavirus Disease-19” OR “COVID 19” OR “COVID 19 Pandemic” OR “COVID 19 Virus Disease” OR “COVID 19 Virus Infection” OR “COVID-19” OR “COVID-19 Pandemic” OR “COVID-19 Pandemics” OR “COVID-19 Virus Disease” OR “COVID-19 Virus Diseases” OR “COVID-19 Virus Infection” OR “COVID-19 Virus Infections” OR “COVID19” OR “Disease 2019, Coronavirus” OR “Disease, 2019-nCoV” OR “Disease, COVID-19 Virus” OR “Infection, 2019-nCoV” OR “Infection, COVID-19 Virus” OR “Infection, SARS-CoV-2” OR “Novel Coronavirus Pneumonia” OR “Pandemic, COVID-19” OR “SARS Coronavirus 2 Infection” OR “SARS CoV 2 Infection” OR “SARS-CoV-2 Infection” OR “SARS-CoV-2 Infections” OR “Severe Acute Respiratory Syndrome Coronavirus 2 Infection” OR “Virus Disease, COVID-19” OR “Virus Infection, COVID-19” OR “Wuhan Coronavirus Epidemic” OR “Wuhan Coronavirus Infection” OR “Wuhan Coronavirus Outbreak” OR “Wuhan Coronavirus Pandemic” OR “Wuhan Coronavirus Pneumonia” OR “Wuhan Seafood Market Pneumonia” |  |
|  | **FS** | **(S1) AND (S2) AND (S3)** |  |
| **Web of Science** |  |  | **126** |
|  | **S1** | “Insulin resistance” OR “Insulin Sensitivity” OR “Resistance, Insulin” OR “Sensitivity, Insulin” |  |
|  | **S2** | “Hyperglycemia” OR “Hyperglycemia, Postprandial” OR “Hyperglycemias” OR “Hyperglycemias, Postprandial” OR “Postprandial Hyperglycemia” OR “Postprandial Hyperglycemias” |  |
|  | **S3** | “2019 nCoV Disease” OR “2019 nCoV Infection” OR “2019 Novel Coronavirus Disease” OR “2019 Novel Coronavirus Epidemic” OR “2019 Novel Coronavirus Infection” OR “2019 Novel Coronavirus Outbreak” OR “2019 Novel Coronavirus Pandemic” OR “2019 Novel Coronavirus Pneumonia” OR “2019-20 China Pneumonia Outbreak” OR “2019-20 Wuhan Coronavirus Outbreak” OR “2019-nCoV Acute Respiratory Disease” OR “2019-nCoV Disease” OR “2019-nCoV Diseases” OR “2019-nCoV Epidemic” OR “2019-nCoV Infection” OR “2019-nCoV Infections” OR “2019-nCoV Outbreak” OR “2019-nCoV Pandemic” OR “2019-nCoV Pneumonia” OR “2019-New Coronavirus Epidemic” OR “2019-Novel Coronavirus (2019-nCoV) Infection” OR “2019-Novel Coronavirus Pneumonia” OR “Coronavirus Disease 19” OR “Coronavirus Disease 2019” OR “Coronavirus Disease-19” OR “COVID 19” OR “COVID 19 Pandemic” OR “COVID 19 Virus Disease” OR “COVID 19 Virus Infection” OR “COVID-19” OR “COVID-19 Pandemic” OR “COVID-19 Pandemics” OR “COVID-19 Virus Disease” OR “COVID-19 Virus Diseases” OR “COVID-19 Virus Infection” OR “COVID-19 Virus Infections” OR “COVID19” OR “Disease 2019, Coronavirus” OR “Disease, 2019-nCoV” OR “Disease, COVID-19 Virus” OR “Infection, 2019-nCoV” OR “Infection, COVID-19 Virus” OR “Infection, SARS-CoV-2” OR “Novel Coronavirus Pneumonia” OR “Pandemic, COVID-19” OR “SARS Coronavirus 2 Infection” OR “SARS CoV 2 Infection” OR “SARS-CoV-2 Infection” OR “SARS-CoV-2 Infections” OR “Severe Acute Respiratory Syndrome Coronavirus 2 Infection” OR “Virus Disease, COVID-19” OR “Virus Infection, COVID-19” OR “Wuhan Coronavirus Epidemic” OR “Wuhan Coronavirus Infection” OR “Wuhan Coronavirus Outbreak” OR “Wuhan Coronavirus Pandemic” OR “Wuhan Coronavirus Pneumonia” OR “Wuhan Seafood Market Pneumonia” OR “SARS-CoV-2” OR “Wuhan Coronavirus” OR “Coronavirus, Wuhan” OR “Wuhan Seafood Market Pneumonia Virus” OR “Coronavirus Disease 2019 Virus” OR “Severe Acute Respiratory Syndrome Coronavirus 2” OR “SARS Coronavirus 2” OR “Coronavirus 2, SARS” OR “COVID19 Virus” OR “COVID19 Viruses” OR “Virus, COVID19” OR “Viruses, COVID19” OR “2019-nCoV” OR “SARS-CoV-2 Virus” OR “SARS CoV 2 Virus” OR “SARS-CoV-2 Viruses” OR “Virus, SARS-CoV-2” OR “COVID-19 Virus” OR “COVID 19 Virus” OR “COVID-19 Viruses” OR “Virus, COVID-19” OR “2019 Novel Coronavirus” OR “2019 Novel Coronaviruses” OR “Coronavirus, 2019 Novel” OR “Novel Coronavirus, 2019” |  |
|  | **FS** | **(S1) AND (S2) AND (S3)** |  |
| **Science Direct** |  |  | **1910** |
|  | **S1** | “Insulin resistance” OR “Insulin Sensitivity” OR “Resistance, Insulin” OR “Sensitivity, Insulin” |  |
|  | **S2** | “Hyperglycemia” OR “Hyperglycemia, Postprandial” OR “Hyperglycemias” OR “Hyperglycemias, Postprandial” OR “Postprandial Hyperglycemia” OR “Postprandial Hyperglycemias” |  |
|  | **S3** | “2019 nCoV Disease” OR “2019 nCoV Infection” OR “2019 Novel Coronavirus Disease” OR “2019 Novel Coronavirus Epidemic” OR “2019 Novel Coronavirus Infection” OR “2019 Novel Coronavirus Outbreak” OR “2019 Novel Coronavirus Pandemic” OR “2019 Novel Coronavirus Pneumonia” OR “2019-20 China Pneumonia Outbreak” |  |
|  | **FS** | **(S1) AND (S2) AND (S3)** |  |
| **Embase** |  |  | **340** |
|  | **S1** | 'resistance, insuline' OR 'insulin resistance' |  |
|  | **S2** | 'glucose blood level, elevated' OR 'glycemia, hyper' OR 'high blood glucose' OR 'hyper-glycaemia' OR 'hyper-glycemia' OR 'hyperglucemia' OR 'hyperglycaemia' OR 'hyperglycaemias' OR 'hyperglycemias' OR 'hyperglycemic syndrome' OR 'hyperglycemia' |  |
|  | **S3** | '2019 novel coronavirus disease' OR '2019 novel coronavirus epidemic' OR '2019 novel coronavirus infection' OR '2019-nCoV disease' OR '2019-nCoV infection' OR 'coronavirus disease 2' OR 'coronavirus disease 2010' OR 'coronavirus disease-19' OR 'coronavirus infection 2019' OR 'COVID' OR 'COVID 19' OR 'COVID 2019' OR 'COVID-10' OR 'COVID-19' OR 'COVID19' OR 'nCoV 2019 disease' OR 'nCoV 2019 infection' OR 'novel coronavirus 2019 disease' OR 'novel coronavirus 2019 infection' OR 'novel coronavirus disease 2019' OR 'novel coronavirus infection 2019' OR 'paucisymptomatic coronavirus disease 2019' OR 'SARS coronavirus 2 infection' OR 'SARS-CoV-2 disease' OR 'SARS-CoV-2 infection' OR 'SARS-CoV2 disease' OR 'SARS-CoV2 infection' OR 'SARSCoV2 disease' OR 'SARSCoV2 infection' OR 'severe acute respiratory syndrome 2' OR 'severe acute respiratory syndrome coronavirus 2 infection' OR 'severe acute respiratory syndrome coronavirus 2019 infection' OR 'severe acute respiratory syndrome CoV-2 infection' OR 'Wuhan coronavirus disease' OR 'Wuhan coronavirus infection' OR 'coronavirus disease 2019' |  |
|  | **FS** | **(S1) AND (S2) AND (S3)** |  |
| **Total records retrieved** | | | **2763** |
