# Supplementary material for: Glycemic dysregulation and insulin resistance across acute and post-acute COVID-19: a systematic review and meta-analysis of etiologic and prognostic evidence
Source: Front Endocrinol (Lausanne). 2026 Oct 14;17:1975728. doi: 10.3389/fendo.2026.1975728 (PMC13713293; doi:10.3389/fendo.2026.1975728)
Supplement: Supplementary file 2 [file Table2.docx]

Supplementary Material

**Supplementary Table 2.** Exposure ascertainment and outcome data for studies included in the prognostic meta-analyses

| **Study** | **Meta-analysis** | **Exposure and comparator definitions** | **Threshold and timing** | **IR or IS assessment** | **Diabetes status** | **Corticosteroid exposure** | **Outcome definition** | **Mortality** **(events/total)** | **IMV** **(events/total)** |
| --- | --- | --- | --- | --- | --- | --- | --- | --- | --- |
| **Montefusco et al. (31)** | Mortality; primary analysis | Study-defined new-onset hyperglycemia versus normoglycemia. Participants with previously known or newly diagnosed diabetes were analyzed separately and were not included in the pooled comparison. | Hyperglycemia was defined in the primary report as one blood glucose measurement between 100 and 199 mg/dL or two measurements >100 and <126 mg/dL. Glycemia was assessed at emergency-department admission, during hospitalization, and at discharge. | HOMA-IR. Continuous glucose monitoring and an arginine-stimulation test were also performed in a selected metabolic subcohort. These assessments did not define the groups used in the meta-analysis. | Known diabetes or newly diagnosed diabetes according to ADA criteria formed a separate group and was excluded from the hyperglycemia-versus-normoglycemia comparison. | Not reported for the prognostic comparison. One patient in the selected metabolic subcohort received corticosteroids. | All-cause mortality within 30 days of hospital admission. | Hyperglycemia: 29/253 Normoglycemia: 14/147 | Not included |
| **Da Porto et al. (32)** | Mortality and IMV; primary analyses | Admission stress hyperglycemia ratio (SHR) >1.14 versus SHR ≤1.14. | SHR was calculated at hospital admission, before intensive insulin treatment, as admission glucose divided by estimated average glucose derived from HbA1c. | HOMA-IR and triglyceride-glucose index. These assessments did not define the exposure groups. | Pre-existing diabetes was permitted and was present in both groups; 56/150 participants had diabetes overall. | Corticosteroid treatment at admission: 1/41 in the SHR >1.14 group and 3/109 in the SHR ≤1.14 group. | All-cause mortality and invasive mechanical ventilation within 60 days of hospital admission. | SHR >1.14: 15/41 SHR ≤1.14: 17/109 | SHR >1.14: 14/41 SHR ≤1.14: 13/109 |
| **Duijvelaar et al. (39)** | Mortality and IMV; primary analyses | Incident grade 3 or higher hyperglycemia, defined by initiation of insulin therapy during hospitalization, versus absence of grade 3 or higher hyperglycemia. | Glucose was measured at admission and daily during hospitalization, with measurements three to four times daily after dexamethasone initiation, in patients with pre-existing diabetes, or when glycemic dysregulation occurred. Insulin was initiated when glucose exceeded 12 mmol/L (approximately 216 mg/dL) or remained consistently above 10 mmol/L (approximately 180 mg/dL). | HOMA-IR was evaluated using glucose and insulin measurements obtained at admission and on day 2 or 3 in a biomarker subset; samples were generally nonfasting. HOMA-IR did not define the exposure groups. | Pre-existing diabetes was permitted and was present in approximately 28% of the cohort. | Dexamethasone was administered during the trial, and glucose monitoring was intensified after its initiation; exposure counts by hyperglycemia group were not reported. | In-hospital all-cause mortality and invasive mechanical ventilation during the index hospitalization. | Grade 3 or higher: 16/59 No grade 3 or higher: 33/326 | Grade 3 or higher: 24/59 No grade 3 or higher: 32/326 |
| **Grubišić et al. (47)** | Mortality and IMV; primary analyses | Study-defined dysglycemia versus normoglycemia. Dysglycemia comprised stress hyperglycemia or newly diagnosed diabetes, distinguished using inpatient glycemic profiles and post-discharge follow-up. | Classification was based on repeated fasting glucose measurements and daily inpatient glycemic profiles. Stress hyperglycemia and newly diagnosed diabetes were adjudicated at 1, 3, and 6 months. For participants who died before follow-up, dysglycemia was assigned when admission fasting glucose was ≥11.1 mmol/L and at least two consecutive inpatient measurements remained elevated. | HOMA-IR measured at hospital admission. HOMA-IR did not define the pooled exposure groups. | Pre-existing diabetes was excluded. Newly diagnosed diabetes was defined by persistent hyperglycemia meeting ADA criteria at 3-month follow-up. | Corticosteroids were administered according to the clinical protocol for hypoxemia; exposure counts by glycemic group were not reported. | In-hospital all-cause mortality and invasive mechanical ventilation during the index hospitalization. | Dysglycemia: 35/72 Normoglycemia: 15/72 | Dysglycemia: 24/72 Normoglycemia: 7/72 |
| **Reiterer et al. (26)** | Mortality and IMV; sensitivity analyses only | Peak blood glucose >170 mg/dL versus peak blood glucose ≤170 mg/dL during hospitalization. | Peak glucose was the highest recorded value during the entire hospitalization. In the mechanistic plasma subcohort, two separate measurements >170 mg/dL were required. | C-peptide and the C-peptide-to-glucose ratio were evaluated in a separate mechanistic subcohort. A ratio >20.5 was used to identify insulin resistance among hyperglycemic patients. These assessments did not define the groups in the full prognostic cohort. | Pre-existing diabetes was permitted and was identified from documented type 1 or type 2 diabetes or HbA1c ≥6.5%. | Corticosteroids were received by 809/3,854 participants overall; exposure according to hyperglycemia group was not reported. | In-hospital all-cause mortality and intubation for invasive mechanical ventilation during the index hospitalization. | Peak glucose >170: 638/1,914 Peak glucose ≤170: 238/1,940 | Peak glucose >170: 684/1,914 Peak glucose ≤170: 67/1,940 |

*Note: Values are presented as events/total participants. Exposure and comparator groups were defined exclusively by study-specific glycemic criteria; insulin-resistance or insulin-sensitivity assessments did not define the participant groups included in the pooled comparisons. Reiterer et al. contributed only to sensitivity analyses because exposure was based on peak glucose recorded at any time during hospitalization. Event counts for Da Porto et al. and Grubišić et al. were reconstructed from reported percentages and group denominators. The classification wording for Montefusco et al. is reproduced as reported in the primary publication.*

*Abbreviations: ADA, American Diabetes Association; HbA1c, glycated hemoglobin; HOMA-IR, homeostatic model assessment of insulin resistance; IMV, invasive mechanical ventilation; IR, insulin resistance; IS, insulin sensitivity; SHR, stress hyperglycemia ratio.*
